# Sumoylation of SAP130 regulates its interaction with FAF1 as well as its protein stability and transcriptional repressor function

**DOI:** 10.1186/s12860-023-00498-x

**Published:** 2024-01-04

**Authors:** Chang-Han Chen, Hung-Wei Lin, Meng-Fang Huang, Chi-Wu Chiang, Kuen-Haur Lee, Nguyen Thanh Phuong, Zong-Yan Cai, Wen-Chang Chang, Ding-Yen Lin

**Affiliations:** 1 Department of Medical Research, Taichung Veterans General Hospital, Taichung, 407219 Taiwan, ROC; 2 Department of Applied Chemistry, and Graduate Institute of Biomedicine and Biomedical Technology, National Chi Nan University, Nantou, 545301 Taiwan, ROC; 3 Department of Biotechnology and Bioindustry Sciences, College of Bioscience and Biotechnology, National Cheng Kung University, Tainan, 70101 Taiwan, ROC; 4 Department of Pharmacology, College of Medicine, National Cheng Kung University, Tainan, 70101 Taiwan, ROC; 5 Institute of Molecular Medicine, College of Medicine, National Cheng Kung University, Tainan, 70101 Taiwan, ROC; 6 Institute for Cancer Biology and Drug Discovery, College of Medical Science and Technology, Taipei Medical University, Taipei, 11031 Taiwan, ROC; 7 Graduate Institute of Medical Sciences, College of Medicine, Taipei Medical University, Taipei Medical University, Taipei, 11031 Taiwan, ROC

**Keywords:** SAP130, FAF1, Sumoylation, SUMO-interacting motifs (SIMs)

## Abstract

**Background:**

Fas-associated factor 1 (FAF1) is a multidomain protein that interacts with diverse partners to affect numerous cellular processes. Previously, we discovered two Small Ubiquitin-like Modifier (SUMO)-interacting motifs (SIMs) within FAF1 that are crucial for transcriptional modulation of mineralocorticoid receptor. Recently, we identified Sin3A-associated protein 130 (SAP130), a putative sumoylated protein, as a candidate FAF1 interaction partner by yeast two-hybrid screening. However, it remained unclear whether SAP130 sumoylation might occur and functionally interact with FAF1.

**Results:**

In this study, we first show that SAP130 can be modified by SUMO1 at Lys residues 794, 878 and 932 both in vitro and in vivo. Mutation of these three SUMO-accepting Lys residues to Ala had no impact on SAP130 association with Sin3A or its nuclear localization, but the mutations abrogated the association of SAP130 with the FAF1. The mutations also potentiated SAP130 trans-repression activity and attenuated SAP130-mediated promotion of cell growth. Additionally, SUMO1-modified SAP130 was less stable than unmodified SAP130. Transient transfection experiments further revealed that FAF1 mitigated the trans-repression and cell proliferation-promoting functions of SAP130, and promoted SAP130 degradation by enhancing its polyubiquitination in a sumoylation-dependent manner.

**Conclusions:**

Together, these results demonstrate that sumoylation of SAP130 regulates its biological functions and that FAF1 plays a crucial role in controlling the SUMO-dependent regulation of transcriptional activity and protein stability of SAP130.

**Supplementary Information:**

The online version contains supplementary material available at 10.1186/s12860-023-00498-x.

## Introduction

Small ubiquitin-like modifiers (SUMOs) are members of the ubiquitin-like superfamily that can be covalently conjugated to various target proteins. This conjugation (sumoylation) is a common, reversible posttranslational modification that serves as a crucial regulatory mechanism in many important biological processes, including transcription, DNA damage response, cell cycle progression, cellular localization, proteostasis, and nuclear body formation (reviewed in [1]). Similar to ubiquitination, sumoylation is an enzymatic reaction catalyzed by a three-enzyme cascade (E1, E2, and E3). Sumoylation of proteins involves the covalent attachment of SUMO to a Lys residue within a short consensus sequence in a target protein (ΨKXD/E, where Ψ is a large hydrophobic residue). In addition to covalent attachment of SUMO to substrate proteins, noncovalent interactions may occur between SUMO and specialized protein domains called SUMO-interacting motifs (SIMs). The most well characterized SIM consists of a hydrophobic core (V/I-X-V/I-V/I or V/I-V/I-X-V/I/L) that is typically flanked by a stretch of acidic residues [2–4]. These non-covalent interactions between SUMO-modified proteins and SIM-containing binding partners may be key contributors to SUMO-dependent functions. For example, SUMO-targeted ubiquitin ligases (STUbLs) are SIM-containing RING finger ubiquitin ligases that recognize poly-sumoylated proteins via a SUMO/SIM interaction and trigger the ubiquitylation and subsequent degradation of the sumolyated targets [5]. Furthermore, recent studies have shown that proteins with both SUMO and ubiquitin conjugates (SUMO-Ub chains) can be recognized by a unique SUMO-Ub chain receptor containing tandem SUMO- and ubiquitin-interacting motifs (tSIM-UIMs) to mediate specific biological functions, such as DNA damage response [6–8]. Thus, defining the functional effects of sumoylation for key protein targets is valuable.

The mSin3A-histone deacetylase corepressor is a multiprotein complex utilized by a wide variety of transcriptional repressors [9, 10]. Sin3A-associated protein 130 (SAP130) was initially identified as a component of the mSin3A corepressor complex by MALDI-TOF mass spectrometry experiments [11]; however, very little is known about the protein. The only predicted motifs in the SAP130 protein are two proline-rich regions, which provides little insight into its potential function within the mSin3A complex. Both mSin3A and HDAC1 are known to bind the C-terminus of SAP130 between amino acids 836 and 1047, but SAP130 lacks a recognizable DNA binding domain. Still, its intrinsic transcriptional repression activity has been demonstrated by utilizing a Gal4-SAP130 chimera to inhibit a Gal4-driven luciferase reporter gene. Additionally, SAP130 has been reported to associate with the Cullin4B-Ring E3 ligase complex (CRL4B), which suggests a role for Sin3A-HDAC complex in CRL4B-mediated transcriptional regulation [12]. Importantly, SAP130 has been found to be modified by SUMO1 [13], but the functional impacts of SAP130 sumoylation remain unclear.

Fas-associated factor 1 (FAF1) was originally identified as a Fas binding partner that potentiates Fas-induced apoptosis [14]. Subsequent research revealed that FAF1 interacts with diverse proteins and is involved in a variety of biological processes. For instance, FAF1 can regulate immune response, as it suppresses NF-kB activity by disrupting IκB kinase (IKK) complex assembly and interfering nuclear translocation of RelA (p65) [15, 16]. Furthermore, FAF1 acts as a positive regulator of type 1 interferon in response to RNA virus infections through its targeting of NLRX1 [17]. Faf1 gene-disrupted mice show embryonic lethality at the two-cell stage [18]. Meanwhile, FAF1 was shown to play a pivotal role in oxidative stress-induced necrosis during the pathogenesis of Parkinson’s disease [19]. Importantly, FAF1 interacts with polyubiquitinated proteins and valosin-containing protein (VCP), which may serve as a scaffold protein, regulating ubiquitin-mediated proteasomal degradation [20]. This FAF1-mediated modulation of protein stability is known to regulate several key cellular processes. For example, FAF1 inhibits Wnt signaling by promoting the proteasomal degradation of β-catenin [21]. Furthermore, FAF1 destabilizes TβRII on the cell surface by recruiting VCP/E3 ligase complex, thereby suppressing TGF-β-mediated cancer metastasis [22]. Along these lines, reduced FAF1 expression was found in some human tumors (reviewed in [23]), making the protein a candidate tumor suppressor.

In previous work, we identified two SIMs within FAF1 and demonstrated that FAF1 interacts with sumoylated mineralocorticoid receptor to represses its transactivation [24]. Notably, two recent studies revealed that C. elegans UBXN-3/FAF1 is an important regulator of DNA replication, as it controls the dynamics of SUMO- and ubiquitin-modified DNA replication factors on chromatin [25, 26]. These studies highlight the critical role of FAF1 for modulating both the SUMO and ubiquitin-modified proteins. Identifying potential novel binding partner(s) of FAF1 may help elucidate the role it plays in controlling protein homeostasis; to do this, we performed yeast two-hybrid screening using FAF1 as bait. Here, we identified SAP130 as a FAF1-interacting protein. In the current study, we sought to determine whether and how the putative interaction between sumoylated SAP130 and FAF1 might occur and affect cellular function.

## Materials and methods

### Plasmids and antibodies

Yeast constructs expressing wild-type (WT) and SIM mutated (DM) Gal AD-FAF, mammalian vectors expressing EGFP-SUMO1, HA-FAF1, Flag-FAF1 WT and DM were described previously [24]. Mammalian expression vectors encoding HA-tagged human SAP130 and HDAC1 (HG17174-NY and HG11486-NY) were purchased from Sino Biological Inc. (Beijing, China). pCS2 + MT-mSin3A was a gift from Bob Eisenman (Addgene plasmid # 30,452; http://n2t.net/addgene:30452; RRID: Addgene_30452). Myc-tagged ubiquitin was cloned into the pCMV-Tga3C vector (Stratagene). A polymerase chain reaction (PCR) fragment encoding the C-terminal of human SAP130 from HA-SAP130 was subcloned into the pBTM116 vector in frame with the LexA domain to generate the LexA-SAP130 bait. SAP130 mutations at three potential SUMO-conjugated Lys residues were created in the pBTM116 and pCMV3 using the Quikchange site-directed mutagenesis kit (Stratagene) with LexA-SAP130, and pCMV3-SAP130 as templates. All constructs were verified by DNA sequencing. The constructs pRS-hMR and pMMTV-Luc were generous gifts from Dr. Ron M. Evans (The Salk Institute, La Jolla, CA). Plasmid M50 Super 8 × TOPFlash was a gift from Randall Moon (Addgene plasmid # 12,456; http://n2t.net/addgene:12456; RRID: Addgene_12456). Plasmid pFR-Luc with five Gal4-binding sites upstream of the minimal promoter driving the luciferase reporter gene and the NF-κB luciferase reporter construct (pNF-κB-Luc) were purchased from Stratagene. The following antibodies were purchased: HA (HA.11; Babco/Covance, Berkley, CA), HA (Biolegend, San Diego, CA), FLAG (M2; Sigma), c-Myc (9E10; GeneTex, Taiwan), GFP (JL-8; BD Biosciences Clontech), FAF1 (C1C3; GeneTex, San Antonio, TX); SAP130 (12130–1-AP, Proteintech), HDAC1 (10E2, Cell Signaling Technology), SIN3A (D9D6, Cell Signaling Technology) and actin (clone AC-74; Sigma, St. Louis, MO).

### Yeast two-hybrid screening and β-Gal assay

Yeast two-hybrid library screening with full-length human FAF1 bait was performed as described previously [24]. Briefly, we transformed the L40 yeast strain with the LexA-FAF1 plasmid followed by transformation with 200 μg of the human testis cDNA library (Clontech). Yeast transformants were then selected on medium containing 5 mM 3-amino-(1,2,4) triazole (Sigma) lacking histidine, leucine, and tryptophan. Histidine protrotophic (His +) colonies were further tested for β-galactosidase activity. The plasmids from both His + and X-gal + colonies were isolated by the curing process of MC1066 bacterial strain and sequenced. The interaction strength was determined according to the appearance of blue color on X-Gal plates, or by the quantitative liquid β-galactosidase assays using lysates from three separate yeast cultures according to the instructions of the Galacto-Light Plus kit (Applied Biosystems).

### Cell culture, transfection and lentivirus-based short hairpin (sh)RNA transduction

HEK293, HEK293T, HeLa, COS-1, HCT116 and L-Wnt3a cells were cultured in Dulbecco's modified Eagle's medium (DMEM; Gibco, Grand Island, NY, USA) supplemented with 10% fetal bovine serum (FBS). NB4 cells were maintained in RPMI 1640 medium (Gibco, Grand Island, NY, USA) containing 10% FBS. The NB4 cell line was kindly gifted from Dr. Tsai-Yun Chen (National Cheng Kung University, Tainan, Taiwan). All other cell lines were obtained from the American Type Culture Collection (Manassas, VA). All cells were incubated at 37 °C in a 5% CO2 atmosphere. Plasmids were transfected using the PolyJet™ reagent (SignaGen Laboratories, Gaithersburg, MD). For stable SAP130 transfection of HEK293 cells, the plasmid constructs of pCMV3-HA-SAP130 WT or 3KA mutant were transfected into HEK293 cells and selected with hygromycin (100 μg/ml). Human FAF1 shRNA (TRCN0000004244) MISSION® shRNA Lentiviral Transduction Particles were purchased from Sigma-Aldrich (St. Louis, MO). To generate lentivirus-shRNA FAF1 knockdown cells, HEK293T, HCT116 and NB4 cells were infected at low confluence (20%) for 24 h with lentiviral supernatants diluted 1:1 with normal culture medium in the presence of 8 μg/ml of polybrene (Sigma). Forty-eight hours after infection, cells transduced by lentiviruses were selected under 2 μg/ml puromycin for 1 week and then passaged before use.

### Growth curves and colony formation assays

The growth rate of cells was monitored by seeding 2 × 10^5^ cells in 60-mm dishes containing 5% FBS. Cells were counted at 24-h intervals using a hemocytometer over a period of seven days. For the colony formation assay, cells were plated at a low density onto 60-mm dishes, and the medium was changed every 3 days. Two weeks later, the number of colonies was counted using the Sigmascan software program after staining with 2% methylene blue.

### Immunoprecipitation and western blotting

For testing the protein–protein interactions in mammalian cells, the expression construct encoding HA-tagged wild-type SAP130 or SAP130 3KA mutant, along with the Flag-tagged FAF1 or myc-tagged mSin3A expression construct, was transfected into COS-1 cells. At 48 h after cotransfection, cells were solubilized in modified RIPA buffer consisting of 50 mM Tris–HCl (pH 7.8), 150 mM NaCl, 5 mM EDTA, 0.5% Triton X-100, 0.5% Nonidet-P40, 0.1% sodium deoxycholate, and a protease inhibitor mixture (Roche Molecular Biochemicals, Indianapolis, IN). To measure the levels of sumoylation, various SAP130 point mutants or wild-type SAP130 and EGFP-SUMO1 constructs were transfected into COS-1 cells. The cells were then lysed with modified RIPA buffer containing 20 mM N-ethylmaleimide (NEM; Sigma). For detection of ubiquitinated SAP130 proteins by immunoprecipitation, HEK293 cells were co-transfected with the plasmid encoding Myc-Ubiquitin and wild-type HA-SAP130, along with plasmids encoding Flag-FAF1 or DM mutant, followed by the treatment with MG132 (5 μM) for another 6 h. Whole cell lysates were mixed with antiserum against HA (Babco/Covance) antibody, and the immunocomplexes were mixed with protein G Plus/protein A-agarose beads (Merck Millipore). After overnight incubation at 4^0^C, the immunocomplexes were then gently washed three times with the PBS buffer followed by Western blot analysis. For Western blot analysis, immunoprecipitated molecules or total cell lysates were separated by SDS-PAGE, transferred onto nitrocellulose membranes (Amersham, GE Healthcare, Germany), blocked with 5% milk, and probed with anti-FLAG or anti-GFP or anti-Myc antibodies. Specific blot signals were visualized on an X-ray film by incubating with ECL chemiluminescence kit (Amersham Biosciences). The specific intensity of each protein band on X-ray film was measured by Image J software (NIH, USA) and results were normalized to corresponding actin band densities.

### Dual-luciferase reporter assay

For reporter gene assays, cells were transfected in 6-well plates with 2 μg of DNA, including the indicated reporter constructs and SAP130 or FAF1 expression vectors, and the constitutive Renilla luciferase control reporter vector pRL-TK (Promega, Madison, WI). The total amount of plasmid per well was kept constant by adding pcDNA3 empty vector. For the experiments of MR activation, cells were treated with either vehicle or 10^−6^ M of aldosterone at 24 h post-transfection, and reporter gene activity was measured after an additional 24 h. To monitor NF-κB transcriptional activity, cells were treated with TNF-α (20 ng/ml) for 6 h at 36 h post-transfection. To stimulate Wnt signaling in HEK293 cells, cells were co-transfected with the TopFlash-Luc reporter, pRL-TK, and HA-FAF1 or SAP130 WT or 3KA mutant plasmids. Cells were treated with Wnt3A conditioned medium (CM) or left untreated and lysed for a luciferase assay at 48 h post-transfection. CM from cells expressing Wnt3A was prepared from L-Wnt3A cells (CRL-2647; American Type Culture Collection Manassas, VA) after 4 days and an additional harvest following 3 days in culture. CM were combined, sterile filtered and mixed 1:1 with normal media for use. Luciferase activity was determined using the dual-luciferase reporter assay system (Promega, Madison, WI).

### Immunofluorescence

HeLa or COS-1 cells were transfected with HA-tagged FAF1, SAP130 WT or 3KA construct by the lipofection method. At 48 h after transfection, the cells were fixed for 10 min with 4% paraformaldehyde in phosphate-buffered saline (PBS) and then permeabilized with cooled acetone for 1 min at -20 °C. The permeabilized cells were then incubated with anti-HA monoclonal antibody alone or combined with SIN3A or HDAC1 antibody for 1 h at room temperature. Subsequently, cells were washed three times with PBS and then incubated with fluorescein isothiocyanate-conjugated anti-mouse or rabbit IgG (Jackson ImmunoResearch) alone or combined with Texas red-conjugated anti-mouse IgG at room temperature for one hour. Nuclei were stained by DAPI (4′,6′-diamidino-2-phenylindole,10 μg/ml). After washing again with PBS, the coverslips were inverted and mounted with GEL/Mount (biomeda corp) to prepare the fluorescence images for analysis with an Olympus BX51 microscope.

### In vitro sumoylation assays

Expression constructs of HA-tagged wild-type or KA mutant SAP130 were transiently transfected into COS-1 cells. Cell extracts were harvested 48 h later for immunoprecipitation with anti-HA antibody and followed by in vitro sumoylation assays. In vitro sumoylation assays were performed as described previously [24]. A typical sumoylation reaction was performed in 20 μl of reaction mixture containing 150 ng of SUMO E1 recombinant proteins (LAE Biotechnology, Taichung, Taiwan), 1 μg of SUMO-1, 1 μg of Ubc9, and proteins bound to beads in a reaction buffer (2 mM ATP, 20 mM HEPES pH 7.5, and 5 mM MgCl_2_). The reactions were carried out at 37 °C for 2 h, and the reactions were stopped by washing with PBS for three times. Beads of samples were analyzed by SDS-PAGE followed by Western blot analysis using anti-HA antibody.

### RNA extraction and real-time quantitative PCR (qPCR)

Total RNA was isolated from control, HEK293 SAP130 WT and 3KA stable cells (approximately 2 × 107) using an RNeasy mini kit (Qiagen, Valencia, CA) and then reverse-transcribed using the ThermoScript RT-PCR system (Invitrogen). The real-time qPCR analysis was performed using the SYBR Green Advantage qPCR Premix (Clontech) and C1000™ Thermal Cycler (Bio-Rad Laboratories, Hercules, CA). PCRs were performed using the following conditions for 30 cycles: 95 °C for 15 s, 60 °C for 15 s, and 72 °C for 20 s. The following primer pairs were used. SAP130, forward (5’-CGGGTCAAAGAGGAGAAGAAA-3’) and reverse (5’-CAGCACAGAGGTGGACTTT-3’), and GAPDH, forward (5’-CCCACTCCTCCACCTTTGAC-3’) and reverse (5’- TCTCTCTTCCTCTTGTGCTCTTG-3’). Relative amounts of the SAP130 transcripts were determined using the comparative CT method and were normalized to the GAPDH housekeeping control.

### cBioPortal analysis

cBioportal website (http://www.cbioportal.org/) is an open-access resource for the interactive exploration of multidimensional cancer genomics data [27, 28]. Using the cBioPortal database, the correlations between mRNA expression of SAP130 and the FAF1 in various cancer types were investigated.

### Statistical analysis

Statistical analyses were performed using two-tailed Student's t-test. P values were calculated using Prism v. 3.03 (GraphPad Software, San Diego, CA).

## Results

### Identification of SAP130 as an FAF1-interacting protein and a SUMO substrate

**Fig. 1:**
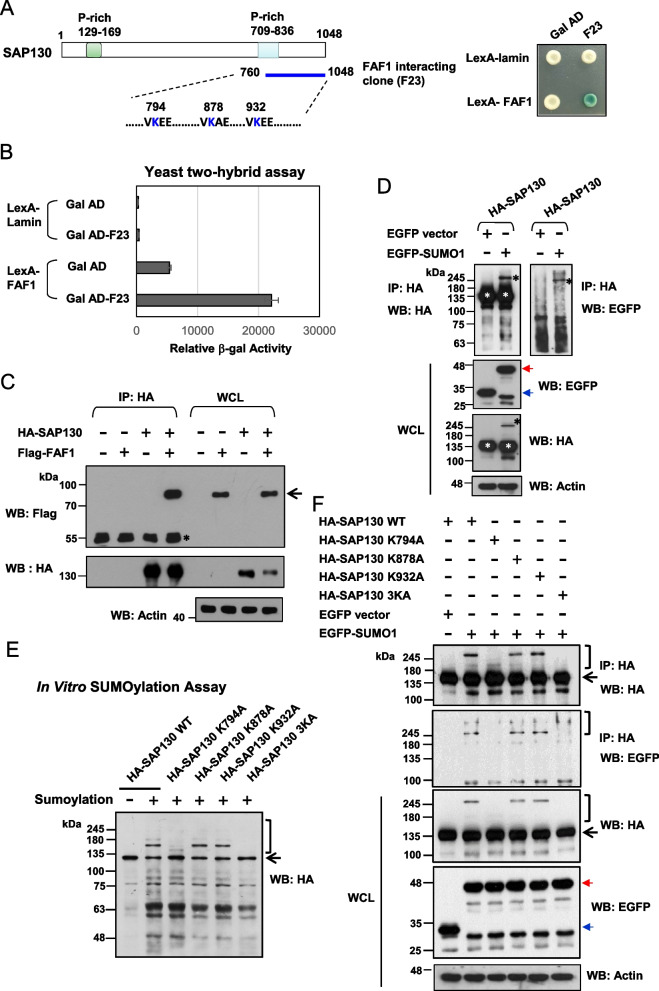
Identification of SAP130 as an FAF1-interacting protein and a SUMO substrate. **A** Schematic representation of the SAP130 clone (F23) that interacted with LexA-FAF1 bait, including the positions of three potential SUMO conjugation sites. **B** Yeast co-transformed as indicated with bait and prey were analyzed with quantitative β-Gal assays and on X-Gal-containing plates. LexA-lamin served as a negative control. **C** COS-1 cells were transiently transfected with the indicated combinations of expression constructs encoding Flag-tagged FAF1 and HA-tagged SAP130, respectively. After 48 h, whole cell lysates (WCL) were used for immunoprecipitation (IP), followed by Western blot (WB) analysis with the indicated antibodies. The arrow indicates the position of the co-precipitated FAF1. Star indicates the heavy chain of IgG. **D** SAP130 is sumoylated in vivo. A plasmid expressing HA-tagged SAP130 was co-transfected with EGFP control or EGFP-SUMO1 plasmid into COS-1 cells. Cell lysates were used for IP with an anti-HA antibody, followed by WB analysis using antibodies against HA and GFP. The black asterisk and white asterisks indicate EGFP-SUMO-1-modified and unmodified SAP130 proteins, respectively. The blue and red arrowheads indicate the EGFP and EGFP-SUMO1 proteins, respectively. **E** SUMO1 modification of SAP130 occurs at Lys-794, 878, and 932. HA-tagged wild-type SAP130 and various mutant SAP130 proteins were subjected to an in vitro sumoylation assay as described in Materials and Methods. SUMO-1 modified and unmodified SAP130 proteins were analyzed by immunoblotting with anti-HA antibody. The bracket and arrow indicate SUMO-modified and unmodified SAP130 proteins, respectively. **F** In vivo sumoylation analyses of wild-type and mutants of SAP130. The assays were performed as described in Fig. 1D. The bracket and arrow indicate EGFP-SUMO-1-modified and unmodified SAP130 proteins, respectively. The blue and red arrowheads indicate the EGFP and EGFP-SUMO1 proteins, respectively. The immunoblots were cropped for clarity. Full length blots are presented in Supplemental Figure S8

We previously conducted a yeast two-hybrid screen using human FAF1 as bait and isolated several candidate FAF1-interacting proteins, including ubiquitin, Ubc9, and SUMO-1 [24]. Among the identified candidates, one clone encoding the C-terminal region of SAP130 spanning amino acid residues 760–1048 was also isolated (Fig. 1A). Interestingly, SAP130 was first identified as a protein that binds to the mSin3A corepressor complex [11]. The specificity of FAF1 association with the clone in yeast cells was validated by quantitative β-gal assays (Fig. 1B). To further verify that SAP130 interacts with FAF1 in mammalian cells, COS-1 cells were co-transfected with expression constructs encoding Flag-FAF1 and HA-SAP130. Cell lysates were subjected to immunoprecipitation with an anti-HA antibody followed by Western blot analysis with an anti-Flag antibody. As shown in Fig. 1C, FAF1 was detected in the immunoprecipitated complexes of SAP130. Furthermore, we noted that the C-terminal fragment of SAP130 isolated by the yeast two-hybrid system contains three putative SUMO conjugation sites at lysine residues K794, K878 and K932, according to the consensus sequence (I/L/V)KXE. While SAP130 was previously reported to be modified by SUMO1 [13], the biological significance of its sumoylation remains unknown. We thus examined whether SAP130 could be modified by SUMO1 in cultured cells transiently expressing HA-SAP130 and EGFP-SUMO1. Immunoprecipitation of HA (SAP130) followed by immunoblotting for either HA or EGFP revealed an additional slower migrating band corresponding to sumoylated SAP130 (Fig. 1D).

We further generated SAP130 mutants in which each lysine residue within the SUMO1 acceptor sites were replaced by alanine (A) or arginine (R), and we evaluated the sumoylation pattern of the mutated proteins by in vitro and in vivo analyses. Our data showed that SAP130 K794A or K794R markedly reduced the extent of SAP130 sumoylation both in vitro and in vivo, suggesting that K794 is a major sumoylation site of SAP130 (Fig. 1E, F and Supplemental Figure S1). Simultaneous mutation of all three lysines in SAP130 (K794A/K878A/K932A; 3KA or K794R/K878R/K932R; 3KR) almost completely abolished the sumoylation of SAP130. These data confirmed that SAP130 is a sumoylation substrate, leading us to hypothesize that FAF1 may interact with sumoylated SAP130.

### SUMO-dependent interaction between FAF1 and SAP130

**Fig. 2:**
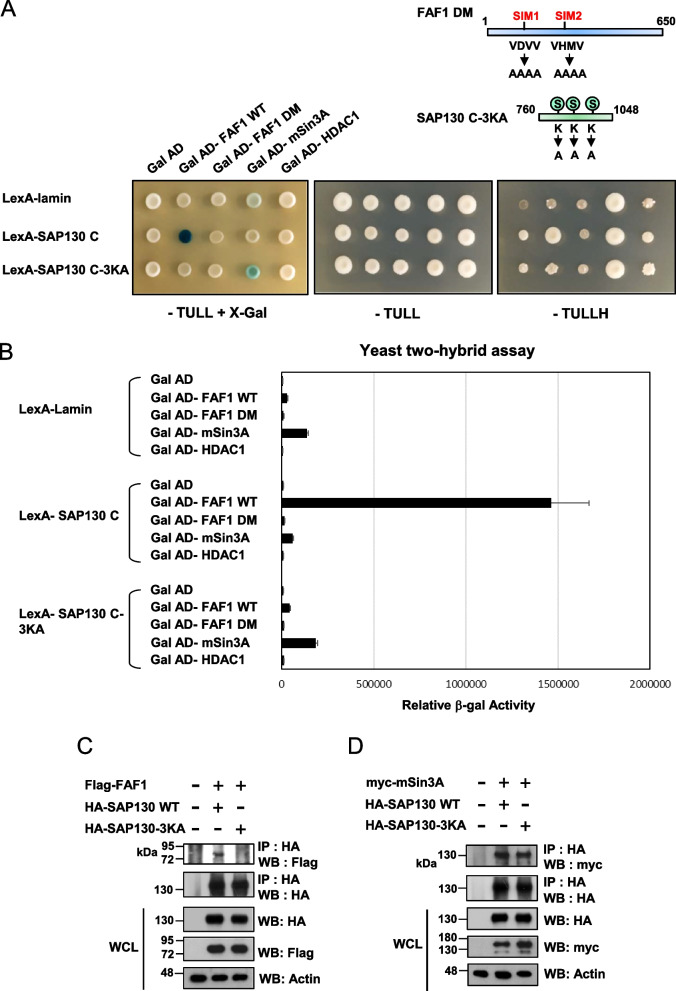
SUMO-dependent interaction between FAF1 and SAP130. **A** Yeast strain L40 was co-transformed with bait (human WT SAP130 or mutant at three sumoylation residues (3KA), or the control protein lamin fused to the LexA DNA-binding domain) and prey constructs [human FAF1 WT, FAF1 with SIM mutant (DM), mSin3A or HDAC1 fused to the Gal-activation domain (AD)]. Yeast transformants were spotted on plates with histidine (-TULL) or without histidine (-TULLH), and with X-Gal (+ X-Gal) media. Schematic presentation of FAF1 DM and SAP130 C-terminal 3KA mutants analyzed in a yeast two-hybrid assay (top). **B** Yeast co-transformed with the indicated bait and prey were analyzed by quantitative β-Gal assays. **C** Coimmunoprecipitation assays. COS-1 cells co-transfected with Flag-FAF1 and HA-SAP130 WT or 3KA mutant of expression constructs were subjected to IP experiments followed by WB analysis with the indicated antibodies. WCL indicates whole cell lysate. **D** COS-1 cells co-transfected with myc-tagged mSin3A and HA-SAP130 WT or 3KA expression constructs were subjected to IP and WB analysis with the indicated antibodies. The immunoblots were cropped for clarity. Full length blots are presented in Supplemental Figure S9

In our previous study, we identified two SIMs within FAF1 and showed that the motifs are crucial for binding for sumoylated mineralocorticoid receptor [24]. Thus, our findings that SAP130 interacts with FAF1 and that SAP130 is sumoylated implied that SUMO might be required for the FAF1-SAP130 interaction. To test this possibility, we constructed WT and 3KA or 3KR mutant SAP130 C-terminal regions (amino acids 760–1048) fused to LexA. Interactions of these constructs with Gal4 AD-FAF1 WT or SIM double mutation (DM) were then assessed for the ability to activate lacZ and HIS3 reporter genes in the yeast two-hybrid system. Since the C- terminal region of SAP130 is also known to bind mSin3A and HDAC1 [11], plasmids expressing full-length mSin3A and HDAC1 fused to the Gal4 AD were also tested in the experiment. Yeast co-transformed with GalAD-FAF1 WT, and LexA-SAP130 C-terminal fragment were able to grow on media lacking histidine and had strong β-gal activity (Fig. 2A), indicating a positive interaction between FAF1 and SAP130. These interactions were further verified by a liquid β-galactosidase assay (Fig. 2B). The interaction between SAP130 and mSin3A was weak as evidenced by the low β-galactosidase activity in the X-gal assay, and no significant interaction could be observed between SAP130 and HDAC1. Remarkably, the SAP130 3KA and 3KR mutants displayed significantly reduced interaction with FAF1 compared to SAP130 WT (Fig. 2A, B and Supplemental Figure S2). Meanwhile, the SAP130 3KA interaction with mSin3A was slightly increased, suggesting that the three lysine residues involved in this interaction. Additionally, the FAF1 DM mutant exhibited markedly reduced binding to SAP130. Together, these results imply that both the sumoylation of SAP130 and SIMs of FAF1 are largely responsible for the interaction between the two proteins.

Next, we performed a reciprocal co-immunoprecipitation assay for SAP130 and FAF1. To do so, we co-transfected Flag-tagged FAF1 and analyzed its association with HA-tagged SAP130 WT or 3KA in COS-1 cells. As shown in Fig. 2C, FAF1 could be coimmunoprecipitated with SAP130 WT, whereas the SAP130 3KA mutant did not readily associate with the FAF1 in this cellular system, indicating that sumoylation is required for the protein–protein interaction. Notably, mutations of the consensus SUMO conjugation sites in SAP130 did not affect the association with mSin3A (Fig. 2D). Together, these results strongly suggest that sumoylation of SAP130 is crucial for binding FAF1.

### Sumoylation of SAP130 protein modulates its transcription-suppressing function, protein stability and cell proliferation-promoting effect

**Fig. 3:**
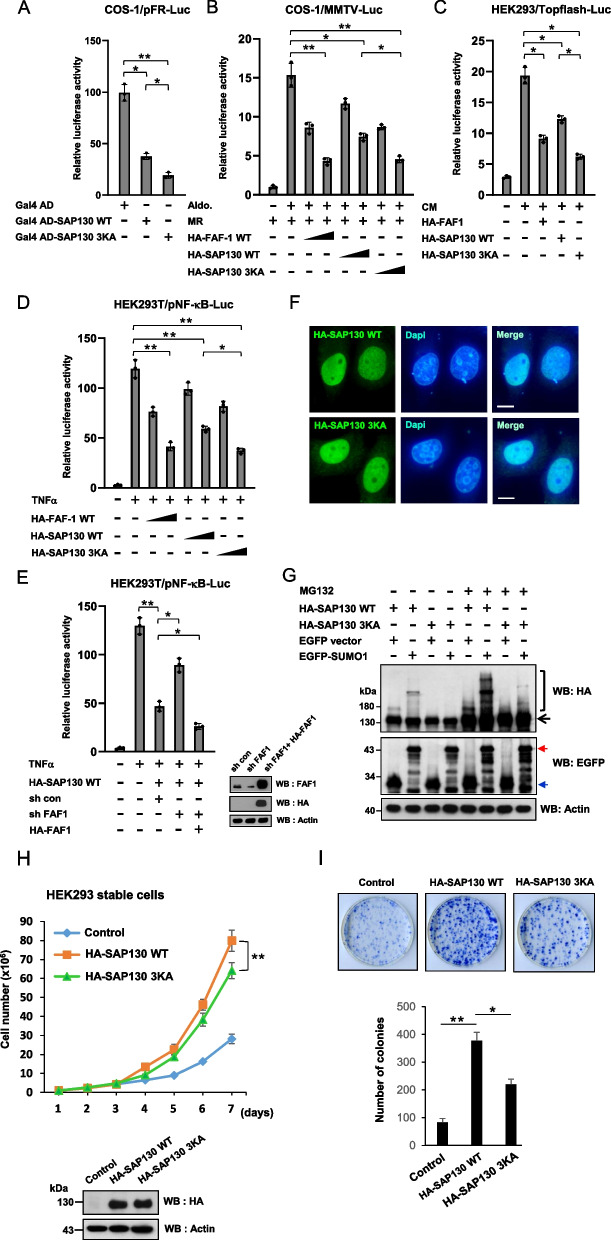
Functional analysis of SAP130 sumoylation. **A** Repression of a Gal4-dependent luciferase reporter gene relative to Gal4 control by Gal4 fusions to SAP130 WT and 3KA mutant. **B** FAF1, SAP130 WT and 3KA mutant efficiently inhibited mineralocorticoid receptor (MR) transactivation. The MMTV-Luc reporter plasmid, the internal control plasmid pRL-TK (Renilla), pRS-hMR, and two different doses of the HA-tagged-FAF1, SAP130 WT or 3KA mutant plasmids were co-transfected into COS-1 cells. The total DNA amount was 2 μg (including addition of empty vector). At 24 h post-transfection, cells were treated with either vehicle or 1 μM aldosterone (Aldo.), and reporter gene activities were measured after another 24 h period. **C** Both SAP130 WT and 3KA mutant could inhibit Wnt signaling. HEK293 cells were co-transfected with the TopFlash-Luc reporter, pRL-TK, and HA-FAF1 or SAP130 WT or 3KA mutant plasmids. Cells were treated with Wnt3A conditioned medium (CM) or left untreated and lysed for a luciferase assay at 48 h post-transfection. **D** Both SAP130 WT and 3KA mutant could efficiently inhibit NF-κB activation. HEK293T cells were co-transfected with pNF-κB-Luc reporter plasmid and pRL-TK plasmid together with two different doses of HA-tagged-FAF1, SAP130 WT or 3KA mutant plasmid. At 36 h post-transfection, cells were treated with tumor necrosis factor (TNF)-α (20 ng/ml) for 6 h or left untreated, and then were subjected to a luciferase assay. **E** SAP130 and FAF1 cooperate in repressing gene transcription. HEK293T cells were transiently transfected with pNF-κB-Luc, SAP130, and FAF1 expression plasmids or infected with lentiviruses expressing control shRNA or FAF1 shRNA as indicated. At 36 h post-transfection/post-transduction, cells were treated with TNF-α (20 ng/ml) for 6 h or left untreated, and followed by a luciferase assay. **F** The SUMO mutation does not alter SAP130 subcellular localization. Immunofluorescence images of HeLa cells transiently expressing HA-tagged SAP130 WT or 3KA as indicated. Immunostaining was performed with an anti-HA antibody (green). DAPI staining shows the position of the nucleus. Scale bars represent 10 μm. **G** Effect of the proteasome inhibitor MG132 on the accumulation of SUMO1-modified and unmodified forms of SAP130. HA-SAP130 WT and 3KA SAP130 mutant were co-transfected with EGFP control or EGFP-SUMO1 plasmid into COS-1 cells. Twenty-four hours after transfection, the cells were cultured in the presence and absence of 5 μM MG132 for another 8 h. SAP130 accumulation was detected by WB with an anti-HA antibody. Cell lysates were also immunoblotted with anti-EGFP antibody to confirm the expression of EGFP and EGFP-SUMO1. The bracket and arrow indicate EGFP-SUMO-1-modified and unmodified SAP130 proteins, respectively. The blue and red arrowheads indicate the EGFP and EGFP-SUMO1 proteins, respectively. **H** Cell growth curves of HEK293 cells stably transfected with constructs expressing control vector, HA-SAP130 WT or 3KA. SAP130 expression was analyzed using immunoblotting. **I** Colony formation assays were performed in stable vector (control), SAP130 WT and SAP130 3KA-overexpressing cells. Colonies were stained with 2% methylene blue 14 days later. Quantitative results in A-E, H and I represent the mean ± SD of three independent experiments. Statistical analyses were performed using two-tailed Student’s t-test. *p < 0.05; **p < 0.01. The immunoblots were cropped for clarity. Full length blots are presented in Supplemental Figure S10

SAP130 is a subunit of the mSin3A*-*histone deacetylase corepressor complex that acts as a transcriptional repressor, and its intrinsic transcriptional repression activity has been demonstrated by testing a Gal4-SAP130 chimera in a Gal4-Luc reporter assay [11]. To assess the effect of SAP130 sumoylation on its transcriptional repression function, we used a similar reporter gene assay in which the DNA binding domain of Gal4 is fused to SAP130 WT or 3KA mutant. COS-1 cells were co-transfected with a luciferase reporter construct (Gal4-Luc) and plasmids encoding Gal4-SAP130 WT or Gal4-SAP130 3KA. The luciferase assay results showed that Gal4-SAP130 3KA exhibited a stronger repression activity than SAP130 WT (Fig. 3A).

The mSin3A corepressor complex has been implicated in transcriptional repression by nuclear hormone receptors and NF-κB signaling pathway [29, 30]. Similarly, studies have shown that FAF1 inhibits mineralocorticoid receptor-mediated transactivation, as well as NF-κB and Wnt/β-catenin signaling. Therefore, we next assessed whether SAP130 sumoylation might play a role in regulating these signaling processes. Our data showed that overexpression of FAF1, SAP130 WT, or SAP130 3KA significantly attenuated transcription of luciferase reporters driven by mineralocorticoid receptor-stimulated MMTV, TNF-α-stimulated NF-κB and Wnt-induced TOPFlash promoters. In all cases, we found that SAP130 WT exhibited a weaker repression activity than FAF1 (Fig. 3B-D). Notably, the SAP130 3KA or 3KR mutant significantly inhibited the reporter activities compared with SAP130 WT, suggesting that the 3KA or 3KR mutant has enhanced repressor activity compared to SAP130 WT (Fig. 3B-D and Supplemental Figure S3). These results imply that sumoylation contributes to the transrepressional function of SAP130. To determine the effects of FAF1 on SAP130-mediated transcriptional repression, we examined whether SAP130-mediated transrepression could be affected by knockdown or overexpression of FAF1. As shown in Fig. 3E, we demonstrated that FAF1 shRNA significantly attenuated SAP130-mediated suppression of TNF-α-stimulated NF-κB promoter. Additionally, overexpression of FAF1 in the FAF1 knockdown background strongly enhanced SAP130-mediated transrepression of NF-κB promoter (Fig. 3E). These results indicate that SAP130 and FAF1 work in concert to regulate gene transcription. Furthermore, the transcriptional repression by SAP130 and FAF1 were not associated with changes in the expression levels and subcellular localization of Sin3A and HDAC1 proteins. We observed that overexpression of FAF1 or SAP130 does not alter the endogenous Sin3A and HDAC1 protein levels and their nuclear localization (Supplemental Figure S4).

It is known that sumoylation is linked to transcriptional repression and events such as changes in subcellular localization, protein half-life and interaction with binding partners, so we tested whether any of these above mentioned events could be involved in altering SAP130 repressor activity. An immunofluorescence analysis in HeLa cells revealed that SAP130 WT, 3KA and 3KR mutant proteins were localized in nucleus (Fig. 3F and Supplemental Figure S5). Thus, SAP130 sumoylation does not appear to be involved in its nuclear targeting. We next tested whether sumoylation controls the stability of SAP130 protein. COS-1 cells were transfected with HA-tagged SAP130 WT or 3KA mutant, followed by treatment with the translation inhibitor cycloheximide (CHX). Cells were then harvested at different time-points and immunoblotted for HA. Interestingly, both proteins showed very similar turnover rate within 3 h (Supplemental Figure S6). However, because only a very small proportion of the SAP130 in the cell is sumoylated at steady state, this method may not reveal the change of stability of sumoylated SAP130. To solve the issue, alternatively, we transfected COS-1 cells with HA-tagged SAP130 WT or 3KA mutant together with EGFP-SUMO1 and then treated with MG132, a proteasome inhibitor. Cells were further lysed and immunoblotted for HA (Fig. 3G). Analyzing and comparing the modified and unmodified SAP130 forms in several experiments revealed that the amount of SUMO1-modified SAP130 was increased to a greater extent in the presence of MG132 (7- to 12-fold increase) than unmodified SAP130 (1.5- to threefold increase). Nevertheless, SAP130 3KA showed an increase in protein levels upon MG132 treatment similar to that of SAP130 WT. These results suggest that SUMO1 modification may potentiate the proteasomal degradation of SAP130, supporting the idea that sumoylation of SAP130 plays a role in its protein stability.

While SAP130 protein has not yet been reported to regulate cell proliferation, we also examined whether sumoylation of SAP130 might regulate cell proliferation. HEK293 cells were stably transfected with SAP130 WT or 3KA mutant and subjected to cell growth analysis. Compared to control cultures, SAP130 WT-overexpressing HEK293 cells showed a significantly increased growth rate over a 7-day period, whereas SAP130 3KA mutant had lower growth-promoting effect than SAP130 WT (Fig. 3H). Additionally, overexpression of SAP130 WT resulted in an increase in colony numbers and sizes compared with vector-transfected cells (Fig. 3I). Consistently, in contrast to SAP130 WT, SAP130 3KA was unable to significant increase the colony formation (Fig. 3I). Together these results suggest that the sumoylation of SAP130 is crucial for regulating its transcription-suppressing function, protein stability and its promotion of cell proliferation.

### FAF1 mitigates SAP130-mediated transrepression and suppresses SAP130-mediated promotion of cell proliferation by promoting SAP130 protein degradation

**Fig. 4:**
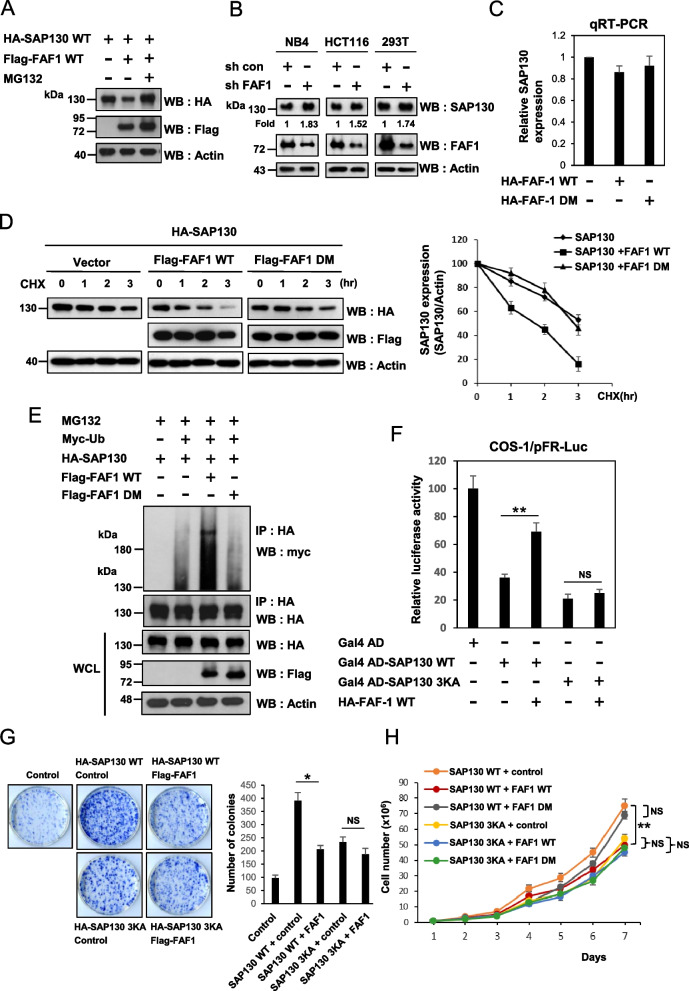
FAF1 motigates SAP130-mediated transrepression and suppresses SAP130-upregulated cell proliferation by promoting SAP130 protein degradation. **A** FAF1 decreases the level of SAP130. HEK293 cells were co-transfected with HA-tagged SAP130 and the Flag-tagged FAF1 WT. At 24 h post-transfection, cells were treated with or without MG132 (5 μM) for 6 h. Cell lysates were immunoblotted with antibodies for HA, Flag and actin. **B** HEK293T, HCT116 and NB4 cells were infected with lentiviruses expressing control non-targeted shRNA or FAF1 shRNA as indicated. Cell lysates were immunoblotted with antibodies for SAP130, FAF1 and actin. **C** FAF1 does not affect the mRNA level of SAP130. The abundance of SAP130 mRNA was measured by RT-qPCR with GAPDH as the internal control. Results represent the mean ± standard deviation of three independent experiments. **D**. COS-1 cells were co-transfected with the HA-SAP130, control vector, Flag-FAF1 WT, or Flag-FAF1 DM. After 48 h, cells were treated with 10 μg/ml cycloheximide (CHX) for the indicated time. Cell lysates were subjected to immunoblotting with an anti-HA or anti-Flag antibody, with actin as a loading control. The SAP130 expression level was quantified, and values were normalized to actin. **E** HEK293 cells were co-transfected with myc-Ub, HA-SAP130 and Flag-FAF1 WT or Flag-FAF1 DM plasmids. After 48 h, cells were treated with MG132 (5 μM) for another 6 h. Cell lysates were used for IP with anti-HA antibody, and bound proteins were detected by WB with anti-HA antibody or anti-myc antibody. The SAP130 and FAF1 levels were determined by immunoblotting. WCL indicates whole cell lysate. **F** Inhibition of SAP130-mediated transcriptional repression by FAF1. COS-1 cells were transiently transfected with Gal4-SAP130 WT or Gal4-SAP130 3KA expression vector in the absence or presence of the indicated FAF1 expression vector together with a Gal4-dependent luciferase reporter. After 48 h, the luciferase activities were measured for each sample. Quantitative results represent the mean ± SD from three independent experiments. **p < 0.01; NS, not significant. **G** and **H** FAF1 suppressed SAP130-mediated cell growth ability. HEK293 cells stably expressing the SAP130 WT or 3KA were transiently transfected with control empty vector or Flag-FAF1 plasmid for 48 h and then split for the cell growth curve and colony formation assays. **G** For the colony formation assay, cells were plated at a low density on dishes. After 2 weeks, cell colonies were stained with methylene blue. **H** Cell growth curve assay demonstrated that overexpressing of FAF1 WT, but not FAF1 DM, inhibited the SAP130-mediated cell proliferation capability on the indicated time points. Quantitative results represent the mean ± SD from three independent experiments. *P < 0.05, **p < 0.01; NS, not significant. The immunoblots were cropped for clarity. Full length blots are presented in Supplemental Figure S11

Up to this point, we had shown that sumoylation of SAP130 is required for its interaction with FAF1 and modulates its protein stability. Since FAF1 acts as a scaffold and to regulate protein degradation, we next tested whether FAF1 affects the steady-state level of SAP130. We first overexpressed FAF1 and observed a consequent decrease in SAP130 protein level (Fig. 4A). Additionally, the FAF1-mediated degradation of SAP130 could be inhibited by pretreatment with the proteasome inhibitor MG132, suggesting that FAF1-mediated SAP130 degradation occurs through a proteasome-dependent pathway (Fig. 4A). In line with these results, knockdown of FAF1 expression in human HEK293T, HCT116 and NB4 cell lines consistently increased endogenous SAP130 protein levels (Fig. 4B and Supplemental Figure S7). Next, we wanted to understand how FAF1 could affect SAP130 protein levels. First, we assessed the impact of FAF1 on the SAP130 mRNA level. Co-transfection of FAF1 WT or FAF1 DM mutant with the SAP130 did not alter SAP130 mRNA levels, suggesting that FAF1 did not affect SAP130 mRNA stability (Fig. 4C). Then, we determined the effects of FAF1 on SAP130 protein half-life. COS-1 cells were co-transfected with either FAF1 WT or DM mutant and then treated with CHX. The SAP130 protein levels at various time-points after CHX treatment were determined by immunoblotting. The results showed that overexpression of FAF1 WT significantly reduced the SAP130 half-life from 3 h to 1.5 h (Fig. 4D). However, overexpression of the DM mutant did not cause a significant change in SAP130 levels. To further test whether and how FAF1 regulates the proteasomal degradation of SAP130, we examined the effects of FAF1 on SAP130 ubiquitination. Overexpression of FAF1 WT significantly increased the ubiquitinated forms of SAP130, but the DM mutant did not (Fig. 4E), suggesting that FAF1 promotes SAP130 degradation by enhancing its polyubiquitination upon SUMO/SIM-mediated binding.

Furthermore, we examined the effects of modulating FAF1 on SAP130-mediated repression of Gal4-responsive luciferase reporter activity. We reasoned that if FAF1 participates in the SAP130 degradation, overexpression of FAF1 should attenuate the repressive effect. As shown in Fig. 3A, co-transfection with the Gal4 luciferase reporter construct with an expression construct for Gal4-SAP130 WT or Gal4-SAP130 3KA mutant could both significantly decreased the reporter activity (Fig. 4F). As expected, co-expression of FAF1 relieved Gal4-SAP130 WT-mediated repressive activity, but it did not affect the activity of Gal4-SAP130 3KA (Fig. 4F). We next explored if FAF1 was involved in regulating SAP130-mediated biological functions, the cell growth ability was assessed. Our data revealed that overexpression of FAF1 WT remarkably attenuated the SAP130 WT-upregulated cell growth by colony formation and cell growth curve assays (Fig. 4G and H). However, FAF1 WT did not have a significant effect on SAP130 3KA-mediated cell growth. Consistently, in contrast to FAF1 WT, FAF1 DM was unable to significant inhibit SAP130 WT and 3KA-mediated cell growth. These findings suggest that FAF1 relieves SAP130-mediated transcriptional repression and pro-proliferative effect in a manner dependent on the SUMO-SIM interactions.

**Fig. 5:**
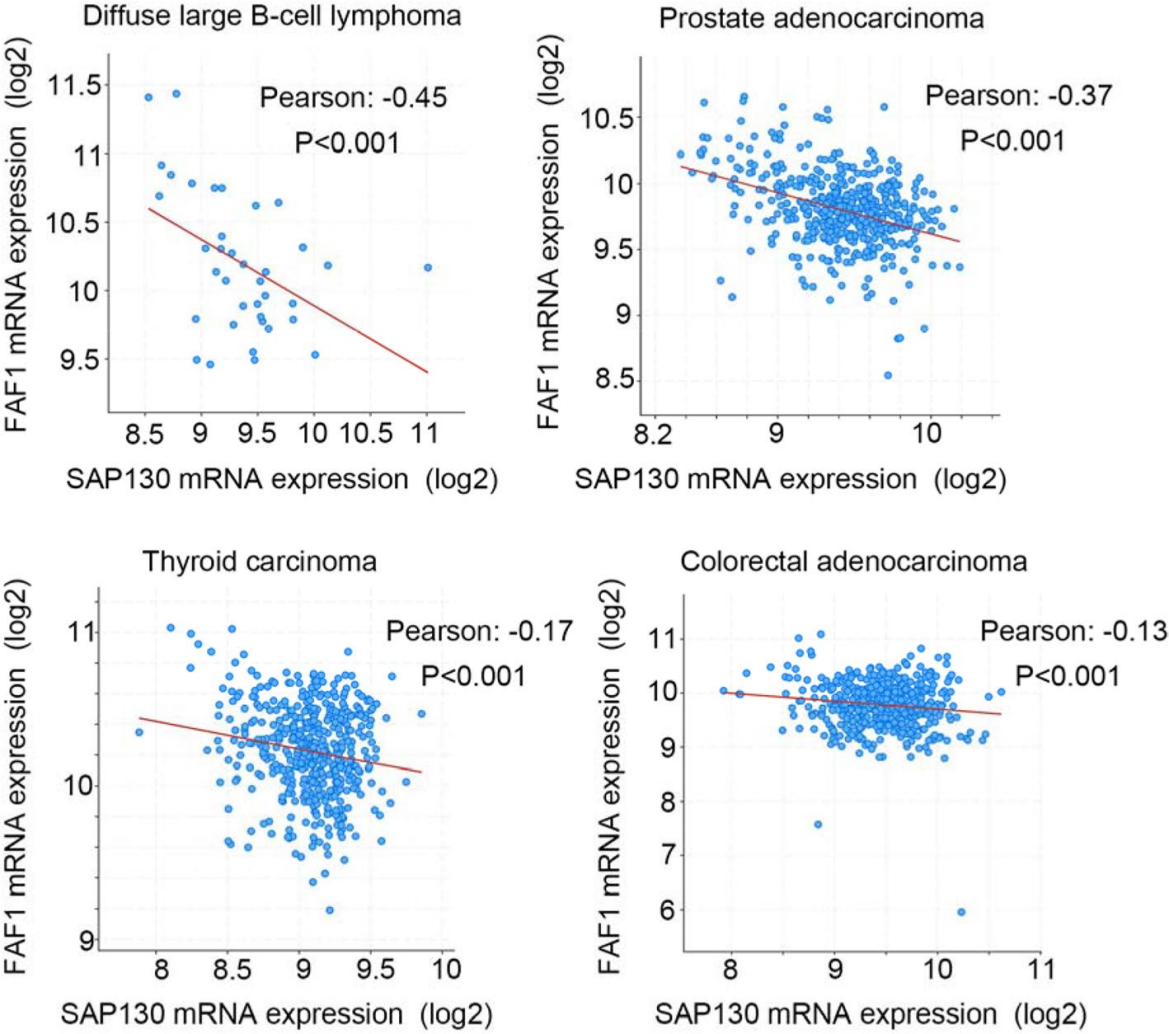
The mRNA levels of SAP130 and FAF1 were negatively correlated in tumor sample from different different types of cancer. Data shown are correlation of the mRNA levels between SAP130 and FAF1 in tumor samples of various types of cancer via cBioPortal database

To further study the relationship between expression of SAP130 and FAF1 in clinical samples, we analyzed the cancer datasets via cBioPortal database. The results indicated that the mRNA level of SAP130 was negatively correlated with that of FAF1 in several types of cancer, such as diffuse large B-cell lymphoma (Person correlation: -0.45; *p* < 0.001), prostate adenocarcinoma (Person correlation: -0.37; *p* < 0.001), thyroid carcinoma (Person correlation: -0.17; *p* < 0.001) and colorectal adenocarcinoma (Person correlation: -0.13; *p* < 0.001) (Fig. 5). These results are consistent with our in vitro data and also suggest that the interaction between SAP130 and FAF1 may play a critical role in cancer development.

## Discussion

In our previous study, we defined two SIMs within FAF1 that are crucial for binding for sumoylated mineralocorticoid receptor [24]. Additionally, we showed that FAF1 can promote the degradation of mineralocorticoid receptor, thereby inhibiting the its transactivation of target genes. Here, we further show that FAF1 physical and functional interaction with SAP130 depends on SIM/SUMO. We first show that SAP130 can be sumoylated at Lys residues 794, 878 and 932. Then, we demonstrate that SAP130 interacts with FAF1 in a SUMO/SIM-dependent manner, but binding of SAP130 to mSin3A/HDAC1 is largely unaffected by sumoylation. We also show that sumoylation of SAP130 inhibits its transrepressive activity, and protein stability but does not affect its nuclear localization. Furthermore, our data suggest that sumoylation is crucial for SAP130-induced promotion of HEK293 cell proliferation and that FAF1 promotes SAP130 ubiquitination and degradation, while downregulating SAP130-mediated transcriptional repression and cell proliferation-promoting function. Thus, our study provides compelling evidence that FAF1 plays an important a regulatory role in SUMO-mediated transcriptional activity and protein stability of its interacting partner SAP130.

Sumoylation is known to affect many characteristics of proteins, including their protein–protein interactions, transcriptional regulation functions, subcellular localization and stability. We defined three SUMO conjugation sites within the SAP130 C-terminal domain and showed that these sites are crucial for interactions with the FAF1. Of note, the C-terminal portion of SAP130 also interacts with mSin3A and HDAC1 [11]. Interestingly, we did not observe evidence for sumoylation-induced changes in the binding of mSin3A to SAP130 (Fig. 2). The association of mSin3A with the SAP130 3KA mutant may also explain why the mutant still exhibits repression activity (Fig. 3A-D). Thus, our findings suggest a conformational or spatial separation between SAP130 structural features involved in its association with mSin3A/HDAC1 complex and those involved in sumoylation-mediated recruitment of FAF1.

We also investigated whether SUMO1 modifications might play a role in modulating the transrepression activity and stability of SAP130. Our results showed that when all three SAP130 SUMO conjugation sites are mutated to Ala, the transcriptional repressive activity is increased compared with WT protein, suggesting that conjugation of SUMO1 to SAP130 attenuates its transcriptional repressor activity (Fig. 3A-D). The mechanism of this change in SAP130 is likely due to sumoylation-mediated alterations in protein stability. Along these lines, we found that SUMO1-modified SAP130 is stabilized to a greater extent than unmodified SAP130 after treatment with MG132 (Fig. 3G), suggesting that SUMO1 modification of SAP130 potentiates proteasomal degradation. We also demonstrated that the interaction between SAP130 and FAF1 promotes proteasome-mediated degradation of SAP130 (Fig. 4E). Consistent with these findings, overexpression of FAF1 significantly inhibited the intrinsic repression ability of SAP130 (Fig. 4F). It is conceivable that a sumoylation-induced interaction with FAF1 may facilitate SAP130 degradation and decrease the transcriptional repressive activity of SAP130. While we provide evidence that SAP130 is a sumoylation target, the physiological determinants regulating its sumoylation remain unknown. It is possible that diverse stimuli participate in this process, and may thereby fine-tune SAP130-regulated transcriptional activity.

Although SAP130 is known to associate with mSin3A/HDAC complex during transcriptional repression, little information exists about the role of SAP130 in the control of cell growth and signaling pathways. Here, we report that sumoylation of SAP130 contributes to transcriptional repression of mineralocorticoid receptor, NF-κB and WNT signaling pathways (Fig. 3B-D). As such, we observed that the SAP130 3KA or 3KR mutant exhibits greater inhibition of these reporter activities than WT SAP130, which suggests a role for SAP130 sumoylation in the regulation of gene transcription. Furthermore, HEK293 cells that stably express SAP130 show enhanced cell proliferation, whereas expression of the SAP130 3KA mutant does not enhance proliferation to the same extent (Fig. 3H and I). Up to now, no role for SAP130 protein in the regulation of cell proliferation has yet been reported, and it still remains unclear how or whether the degrees of SAP130 expression and sumoylation may be associated with tumorigenesis. It will therefore be of interest to further clarify whether SAP130 has tumor-promoting potential. Given that FAF1 has been implicated as a tumor-suppressor and we demonstrated that FAF1 inhibits SAP130-mediated transcriptional function, stability and cell proliferation-promoting ability, it is possible that FAF1 would attenuate any oncogenic potential of SAP130. Notably, analysis of the cancer datasets via cBioPortal database revealed that the mRNA level of SAP130 was negative correlated with FAF1 mRNA level in several types of cancers (Fig. 5). Thus, our findings warrant new studies to expand our understanding of the roles of SAP130 and FAF1 in carcinogenesis.

FAF1 participates the proteasomal degradation of ubiquitinated proteins, as it is thought to act as a scaffold protein or ubiquitin receptor for multiple ubiquitin-related domains, including ubiquitin-associated (UBA), ubiquitin-like 1 and 2 (UBL1, UBL2), and ubiquitin-regulatory X (UBX) domains [20]. Our work provides novel mechanistic insights into the role of FAF1/SIMs in SUMO-dependent transcriptional modulation and proteolysis ([24] and this study). Recent studies have revealed the importance of SUMO-Ub chain receptors that contain tandem SIMs and UIMs (tSIM-UIMs) [6–8]. In addition, two very recent studies reported that *C. elegans* UBXN-3/FAF1 is an important coordinator of DNA replication, as it controls the turnover of SUMO- and ubiquitin-modified DNA replication factors [25, 26]. Taken together, these studies and ours highlight the importance of FAF1 in modulating the stability of SUMO- and ubiquitin-modified proteins. Further work will be required to clarify how the SIMs and ubiquitin-related domains of FAF1 differentially contribute to its regulation of diverse biological processes.

### Supplementary Information

**Additional file 1:** **Supplementary Figure S1.** Characterization of SUMO target lysine residues in SAP130. Western blot showing HA-tagged SAP130 modified by EGFP-SUMO-1. A plasmid expressing HA-tagged wild-type SAP130 or various mutant SAP130 was co-transfected with EGFP control or EGFP-SUMO1 plasmid into COS-1 cells. Cell lysates were used for WB analysis using antibodies against HA and EGFP. The bracket and arrow indicate EGFP-SUMO-1-modified and unmodified SAP130 proteins, respectively. The blue and red arrowheads indicate the EGFP and EGFPSUMO1 proteins, respectively. Notably, the mutation of Lysine (K)-794, 878, and 932 to alanine (A) or arginine (R) showed the similar results. **Supplementary Figure S2.** SUMO-dependent interaction between FAF1 and SAP130. (A) Yeast strain L40 was co-transformed with bait (human WT SAP130 or mutant at three sumoylation residues (3KA or 3KR), or the control protein lamin fused to the LexA DNA-binding domain) and prey constructs [human FAF1 WT or FAF1 with SIM mutant (DM) fused to the Gal-activation domain (AD)]. Yeast transformants were spotted on plates with histidine (-TULL) or without histidine (-TULLH), and with X-Gal (+X-Gal) media. Schematic presentation of FAF1 DM, SAP130 C-terminal 3KA and 3KR mutants analyzed in a yeast two-hybrid assay (top). (B) Yeast co-transformed with the indicated bait and prey were analyzed by quantitative β-Gal assays. **Supplementary Figure S3.** Sumoylation of SAP130 protein modulates its transcription-suppressing function. The SAP130 3KA or 3KR mutant exhibited similar transcriptional repression activity on MR-stimulated MMTV and TNF-α-stimulated NF-κB luciferase reporters. (A) The MMTV-Luc reporter plasmid, the internal control plasmid pRL-TK (Renilla), pRS-hMR, and HA-tagged-SAP130 WT, 3KA or 3KR mutant plasmids were co-transfected into COS-1 cells. The total DNA amount was 2 μg (including addition of empty vector). At 24 h post-transfection, cells were treated with either vehicle or 1 μM aldosterone (Aldo.), and reporter gene activities were measured after another 24 h period. (B) HEK293T cells were co-transfected with pNF-κB-Luc reporter plasmid and pRL-TK plasmid together with HA-tagged-SAP130 WT, 3KA or 3KR mutant plasmid. At 36 h post-transfection, cells were treated with tumor necrosis factor (TNF)-α (20 ng/ml) for 6 h or left untreated, and then were subjected to a luciferase assay. Results represent the mean ± SD of three independent experiments. Statistical analyses were performed using two-tailed Student’s t-test. *p < 0.05. **Supplementary Figure S4.** Overexpression of FAF1 or SAP130 does not alter the endogenous Sin3A and HDAC1 protein levels and their nuclear localization. (A) HEK293T cells were transfected with the control vector or two different doses of the HA-tagged-FAF1, SAP130 WT or 3KA mutant plasmid. At 48 h post-transfection, cell lysates were subjected to immunoblotting with an anti-HA , Sin3A or HDAC1 antibody, with actin as a loading control. The immunoblots were cropped for clarity. Full length blots are presented in Supplemental Figure S12. (B) COS-1 cells were transiently transfected with HA-tagged FAF1, SAP130 WT or 3KA expression plasmid. After 48 h, cells were subjected to immunostaining analyses with anti-HA antibody (green) and anti-Sin3A or HDAC1 antibody (red). The secondary antibodies for these studies were fluorescein isothiocyanateconjugated anti-rabbit IgG and Texas red-conjugated anti-mouse IgG. DAPI staining shows the position of the nucleus. The open arrowhead indicates the HA-FAF1 or SAP130-transfected cells. **Supplementary Figure S5.** The SUMO mutation does not alter SAP130 subcellular localization. Immunofluorescence images of HeLa cells transiently expressing HA-tagged SAP130 WT, 3KA or 3KR as indicated. Immunostaining was performed with an anti-HA antibody (green). DAPI staining shows the position of the nucleus. **Supplementary Figure S6.** COS-1 cells were transfected with the HA-SAP130 WT or HA-SAP130 3KA. After 48 h, cells were treated with 10 μg/ml cycloheximide (CHX) for the indicated time. Cell lysates were subjected to immunoblotting with an anti-HA antibody, with actin as a loading control. The SAP130 expression level was quantified, and values were normalized to actin. The immunoblots were cropped for clarity. Full length blots are presented in Supplemental Figure S13. **Supplementary Figure S7.** HEK293T, HCT116 and NB4 cells were infected with lentiviruses expressing control non-targeted shRNA or FAF1 shRNA as indicated. Cell lysates were immunoblotted with antibodies for SAP130, FAF1 and actin as in Fig. 4B. Quantification of endogenous SAP130 protein levels in shFAF1 lentiviruses infected NB4 (A), HCT116 (B) and HEK293T cells (C). Three independent Western Blots were quantified by densitometry using Image J software. The protein expression of SAP130 was normalized with respect to the corresponding actin band densities. *p < 0.05; **p < 0.01. **Supplementary Figure S8.** Full length blots used in Fig. 1. The cropped areas used in Fig. 1 are shown in red boxes. **Supplementary Figure S9.** Full length blots used in Fig. 2. The cropped areas used in Fig. 2 are shown in red boxes. **Supplementary Figure S10.** Full length blots used in Fig. 3. The cropped areas used in Fig. 3 are shown in red boxes. **Supplementary Figure S11.** Full length blots used in Fig. 4. The cropped areas used in Fig. 4 are shown in red boxes. **Supplementary Figure S12.** Full length blots used in supplementary Figure S4. The cropped areas used in supplementary Figure S4 are shown in red boxes. **Supplementary Figure S13.** Full length blots used in supplementary Figure S6. The cropped areas used in supplementary Figure S6 are shown in red boxes.

## Data Availability

The reagents used and data generated in this study are available from the corresponding author on reasonable request.
