# Supplementary material for: Sumoylation of SAP130 regulates its interaction with FAF1 as well as its protein stability and transcriptional repressor function
Source: BMC Mol Cell Biol. 2024 Jan 4;25:2. doi: 10.1186/s12860-023-00498-x (PMC10765799; doi:10.1186/s12860-023-00498-x)
Supplement: Supplementary file 1 — Additional file 1: Supplementary Figure S1. Characterization of SUMO target lysine residues in SAP130. Western blot showing HA-tagged SAP130 modified by EGFP-SUMO-1. A plasmid expressing HA-tagged wild-type SAP130 or various mutant SAP130 was co-transfected with EGFP control or EGFP-SUMO1 plasmid into COS-1 cells. Cell lysates were used for WB analysis using antibodies against HA and EGFP. The bracket and arrow indicate EGFP-SUMO-1-modified and unmodified SAP130 proteins, respectively. The blue and red arrowheads indicate the EGFP and EGFPSUMO1 proteins, respectively. Notably, the mutation of Lysine (K)-794, 878, and 932 to alanine (A) or arginine (R) showed the similar results. Supplementary Figure S2. SUMO-dependent interaction between FAF1 and SAP130. (A) Yeast strain L40 was co-transformed with bait (human WT SAP130 or mutant at three sumoylation residues (3KA or 3KR), or the control protein lamin fused to the LexA DNA-binding domain) and prey constructs [human FAF1 WT or FAF1 with SIM mutant (DM) fused to the Gal-activation domain (AD)]. Yeast transformants were spotted on plates with histidine (-TULL) or without histidine (-TULLH), and with X-Gal (+X-Gal) media. Schematic presentation of FAF1 DM, SAP130 C-terminal 3KA and 3KR mutants analyzed in a yeast two-hybrid assay (top). (B) Yeast co-transformed with the indicated bait and prey were analyzed by quantitative β-Gal assays. Supplementary Figure S3. Sumoylation of SAP130 protein modulates its transcription-suppressing function. The SAP130 3KA or 3KR mutant exhibited similar transcriptional repression activity on MR-stimulated MMTV and TNF-α-stimulated NF-κB luciferase reporters. (A) The MMTV-Luc reporter plasmid, the internal control plasmid pRL-TK (Renilla), pRS-hMR, and HA-tagged-SAP130 WT, 3KA or 3KR mutant plasmids were co-transfected into COS-1 cells. The total DNA amount was 2 μg (including addition of empty vector). At 24 h post-transfection, cells were treated with either vehicle or 1 μM aldosterone [file 12860_2023_498_MOESM1_ESM.pdf]

Sumoylation of SAP130 regulates its interaction with FAF1 as well as its protein stability and transcriptional repressor function

Chang-Han Chen, Hung-Wei Lin, Meng-Fang Huang, Chi-Wu Chiang, Kuen-Haur Lee, Nguyen Thanh Phuong, Zong-Yan Cai , Wen-Chang Chang and Ding-Yen Lin

Supplemental Information

Supplemental Figure S1

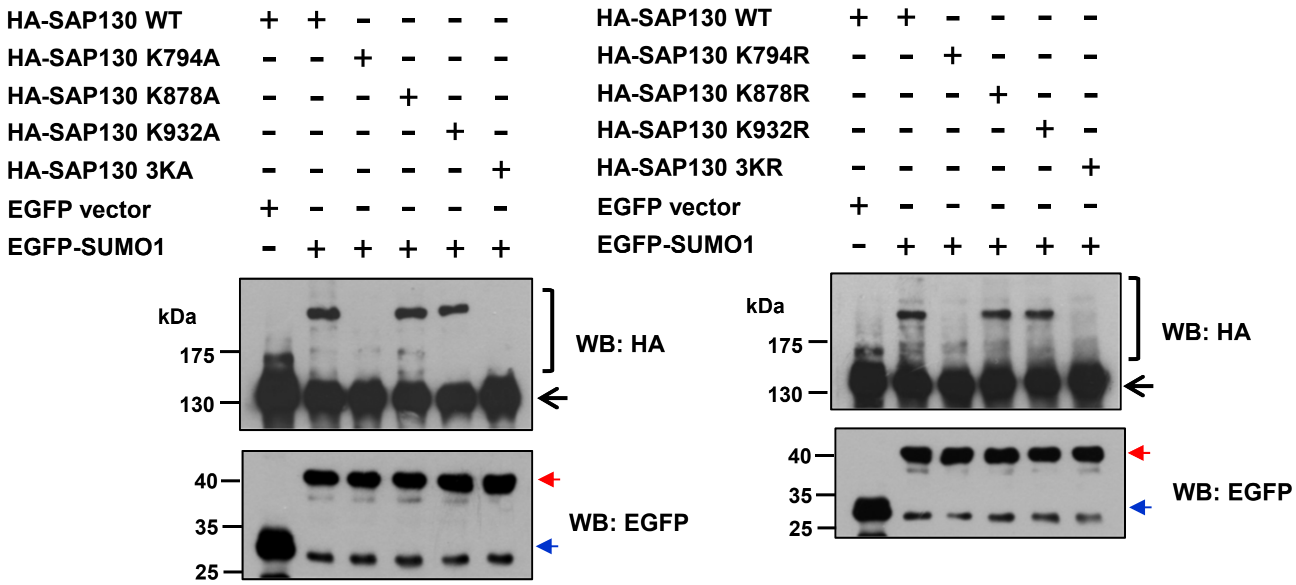

Supplementary Figure S1

Characterization of SUMO target lysine residues in SAP130. Western blot showing HA-tagged SAP130 modified by EGFP-SUMO-1. A plasmid expressing HA-tagged wild-type SAP130 or various mutant SAP130 was co-transfected with EGFP control or EGFP-SUMO1 plasmid into COS-1 cells. Cell lysates were used for WB analysis using antibodies against HA and EGFP. The bracket and arrow indicate EGFP-SUMO-1-modified and unmodified SAP130 proteins, respectively. The blue and red arrowheads indicate the EGFP and EGFP-SUMO1 proteins, respectively. Notably, the mutation of Lysine (K)-794, 878, and 932 to alanine (A) or arginine (R) showed the similar results.

Supplemental Figure S2

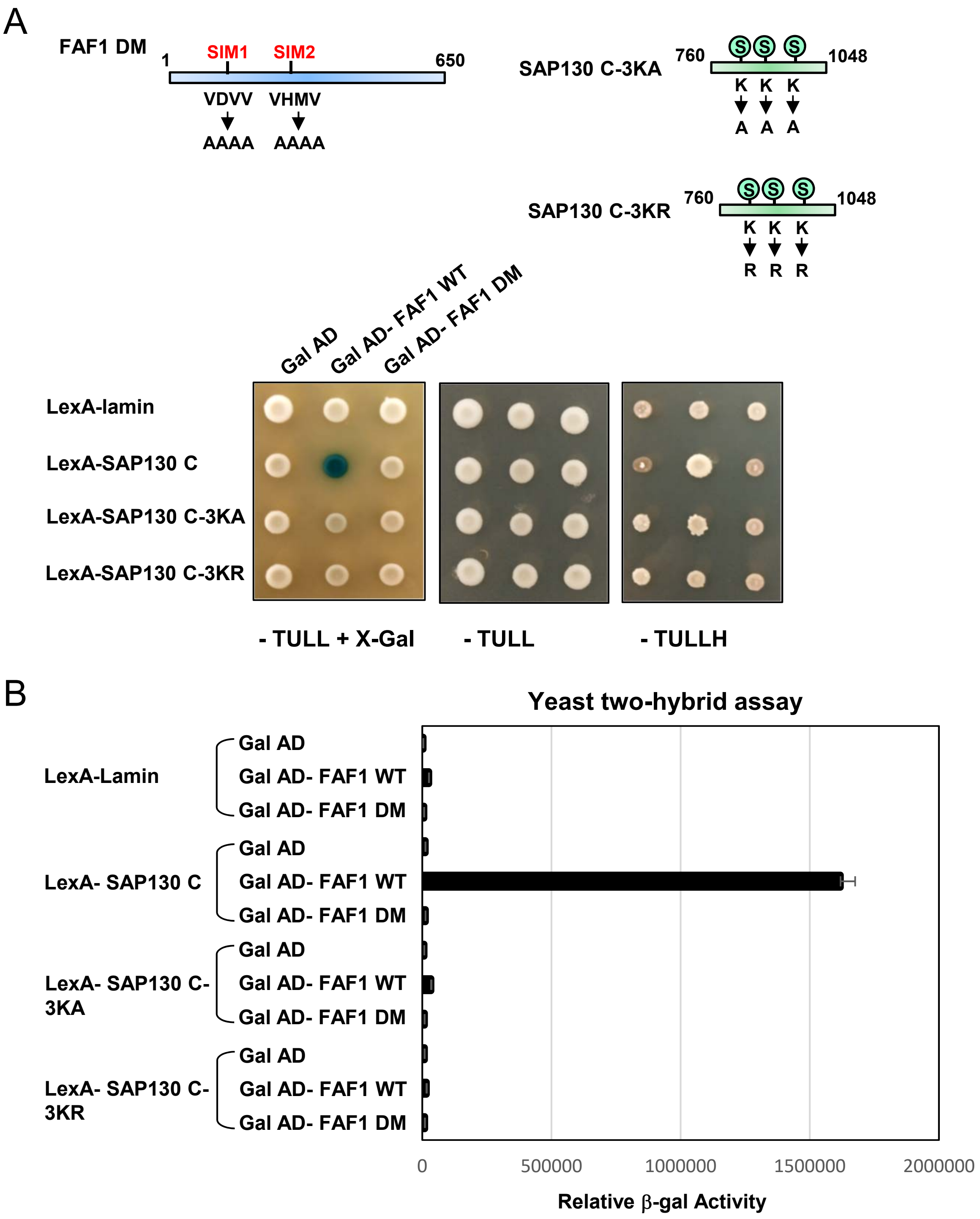

Supplementary Figure S2. SUMO-dependent interaction between FAF1 and SAP130. (A) Yeast strain L40 was co-transformed with bait (human WT SAP130 or mutant at three sumoylation residues (3KA or 3KR), or the control protein lamin fused to the LexA DNA-binding domain) and prey constructs [human FAF1 WT or FAF1 with SIM mutant (DM) fused to the Gal-activation domain (AD)]. Yeast transformants were spotted on plates with histidine (-TULL) or without histidine (-TULLH), and with X-Gal (+X-Gal) media. Schematic presentation of FAF1 DM, SAP130 C-terminal 3KA and 3KR mutants analyzed in a yeast two-hybrid assay (top). (B) Yeast co-transformed with the indicated bait and prey were analyzed by quantitative β-Gal assays.

Supplemental Figure S3

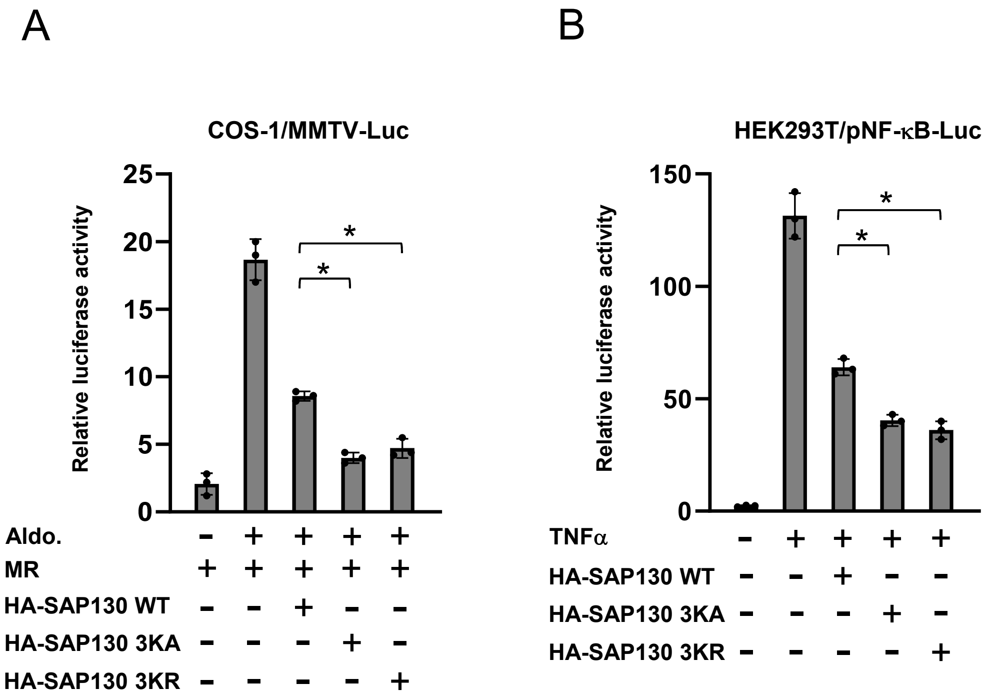

Supplementary Figure S3

Sumoylation of SAP130 protein modulates its transcription-suppressing function. The SAP130 3KA or 3KR mutant exhibited similar transcriptional repression activity on MR-stimulated MMTV and TNF-α-stimulated NF-κB luciferase reporters. (A) The MMTV-Luc reporter plasmid, the internal control plasmid pRL-TK (Renilla), pRS-hMR, and HA-tagged-SAP130 WT, 3KA or 3KR mutant plasmids were co-transfected into COS-1 cells. The total DNA amount was 2 μg (including addition of empty vector). At 24 h post-transfection, cells were treated with either vehicle or 1 μM aldosterone (Aldo.), and reporter gene activities were measured after another 24 h period. (B) HEK293T cells were co-transfected with pNF-κB-Luc reporter plasmid and pRL-TK plasmid together with HA-tagged-SAP130 WT, 3KA or 3KR mutant plasmid. At 36 h post-transfection, cells were treated with tumor necrosis factor (TNF)-α (20 ng/ml) for 6 h or left untreated, and then were subjected to a luciferase assay. Results represent the mean ± SD of three independent experiments. Statistical analyses were performed using two-tailed Student's t-test. *$p < 0.05$.

Supplemental Figure S4

A

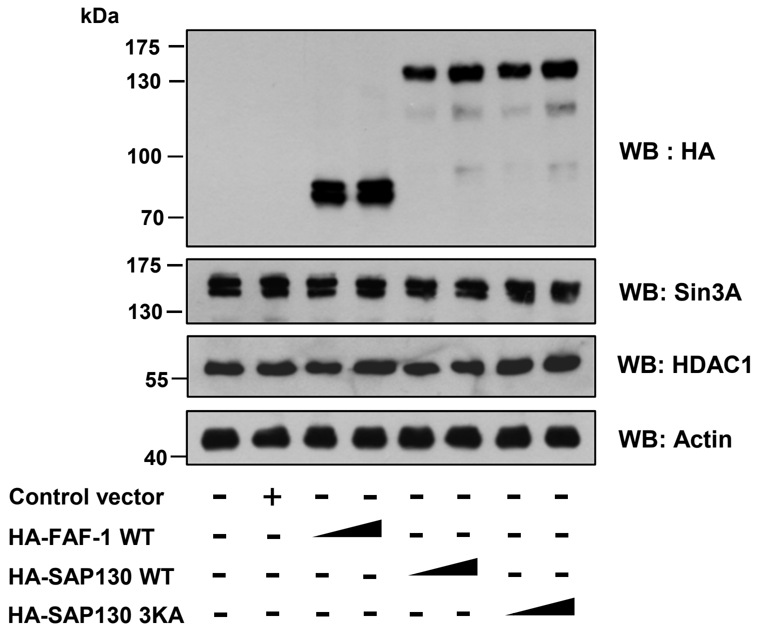

B

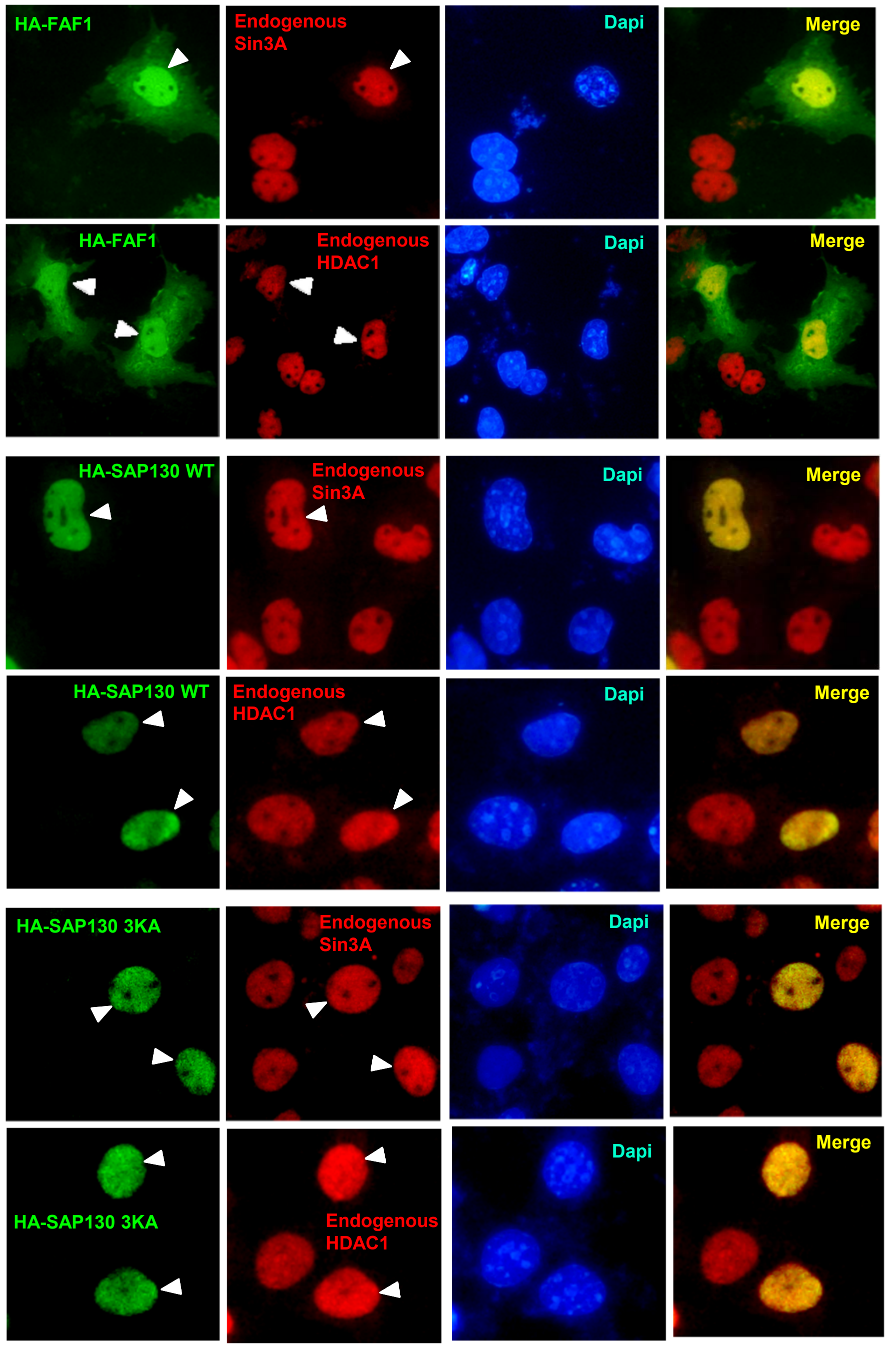

Supplementary Figure S4.

Overexpression of FAF1 or SAP130 does not alter the endogenous Sin3A and HDAC1 protein levels and their nuclear localization.

(A) HEK293T cells were transfected with the control vector or two different doses of the HA-tagged-FAF1, SAP130 WT or 3KA mutant plasmid. At 48 h post-transfection, cell lysates were subjected to immunoblotting with an anti-HA , Sin3A or HDAC1 antibody, with actin as a loading control. The immunoblots were cropped for clarity. Full length blots are presented in Supplemental Figure S12. (B) COS-1 cells were transiently transfected with HA-tagged FAF1, SAP130 WT or 3KA expression plasmid. After 48 h, cells were subjected to immunostaining analyses with anti-HA antibody (green) and anti-Sin3A or HDAC1 antibody (red). The secondary antibodies for these studies were fluorescein isothiocyanate-conjugated anti-rabbit IgG and Texas red-conjugated anti-mouse IgG. DAPI staining shows the position of the nucleus. The open arrowhead indicates the HA-FAF1 or SAP130-transfected cells.

Supplemental Figure S5

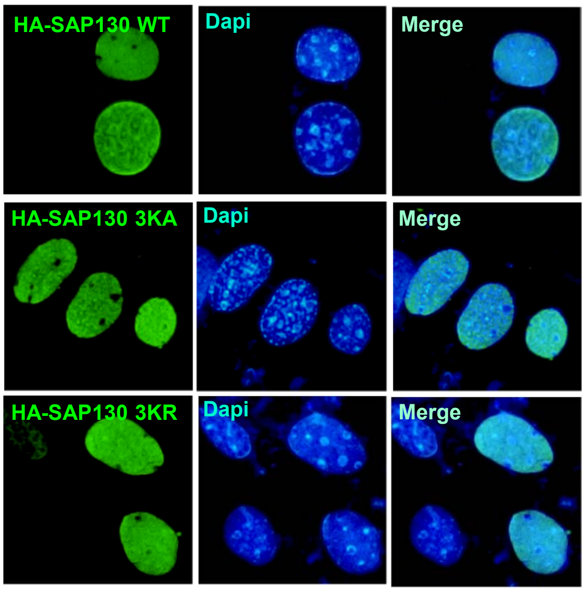

Supplementary Figure S5

The SUMO mutation does not alter SAP130 subcellular localization. Immunofluorescence images of HeLa cells transiently expressing HA-tagged SAP130 WT, 3KA or 3KR as indicated. Immunostaining was performed with an anti-HA antibody (green). DAPI staining shows the position of the nucleus.

Supplemental Figure S6

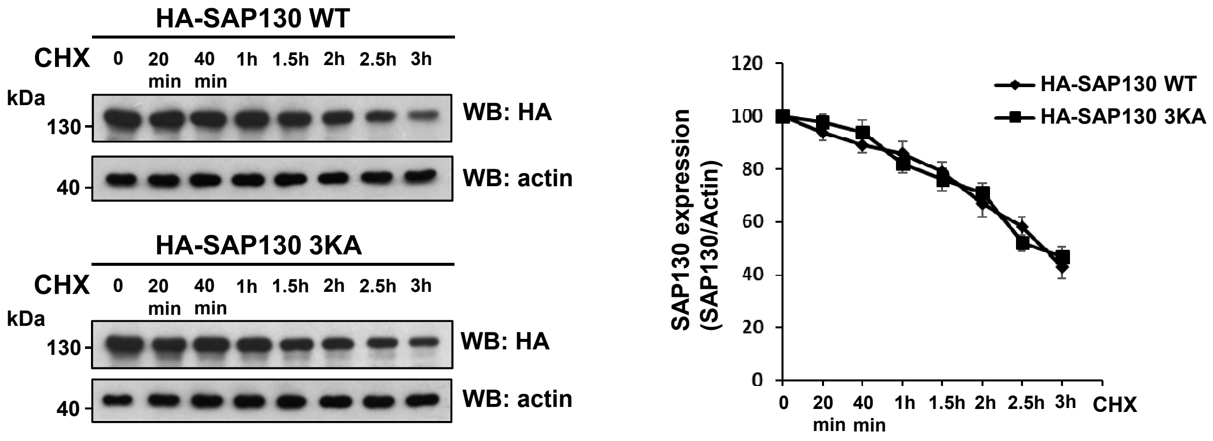

Supplementary Figure S6. COS-1 cells were transfected with the HA-SAP130 WT or HA-SAP130 3KA. After 48 h, cells were treated with 10 µg/ml cycloheximide (CHX) for the indicated time. Cell lysates were subjected to immunoblotting with an anti-HA antibody, with actin as a loading control. The SAP130 expression level was quantified, and values were normalized to actin. The immunoblots were cropped for clarity. Full length blots are presented in Supplemental Figure S13.

Supplemental Figure S7

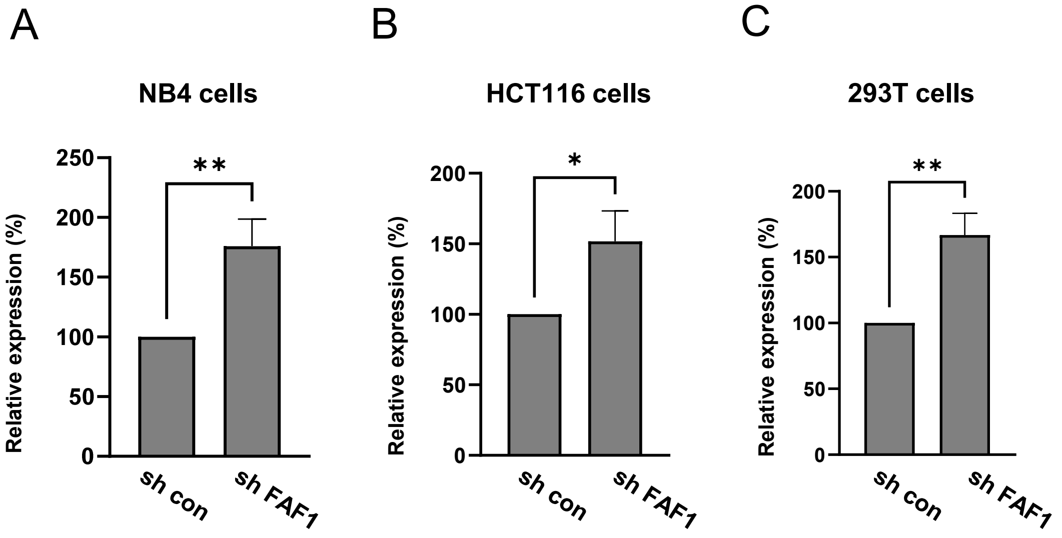

Supplementary Figure S7.
HEK293T, HCT116 and NB4 cells were infected with lentiviruses expressing control non-targeted shRNA or FAF1 shRNA as indicated. Cell lysates were immunoblotted with antibodies for SAP130, FAF1 and actin as in Figure 4B. Quantification of endogenous SAP130 protein levels in shFAF1 lentiviruses infected NB4 (A), HCT116 (B) and HEK293T cells (C). Three independent Western Blots were quantified by densitometry using Image J software. The protein expression of SAP130 was normalized with respect to the corresponding actin band densities. *p < 0.05; **p < 0.01.

Supplemental Figure S8

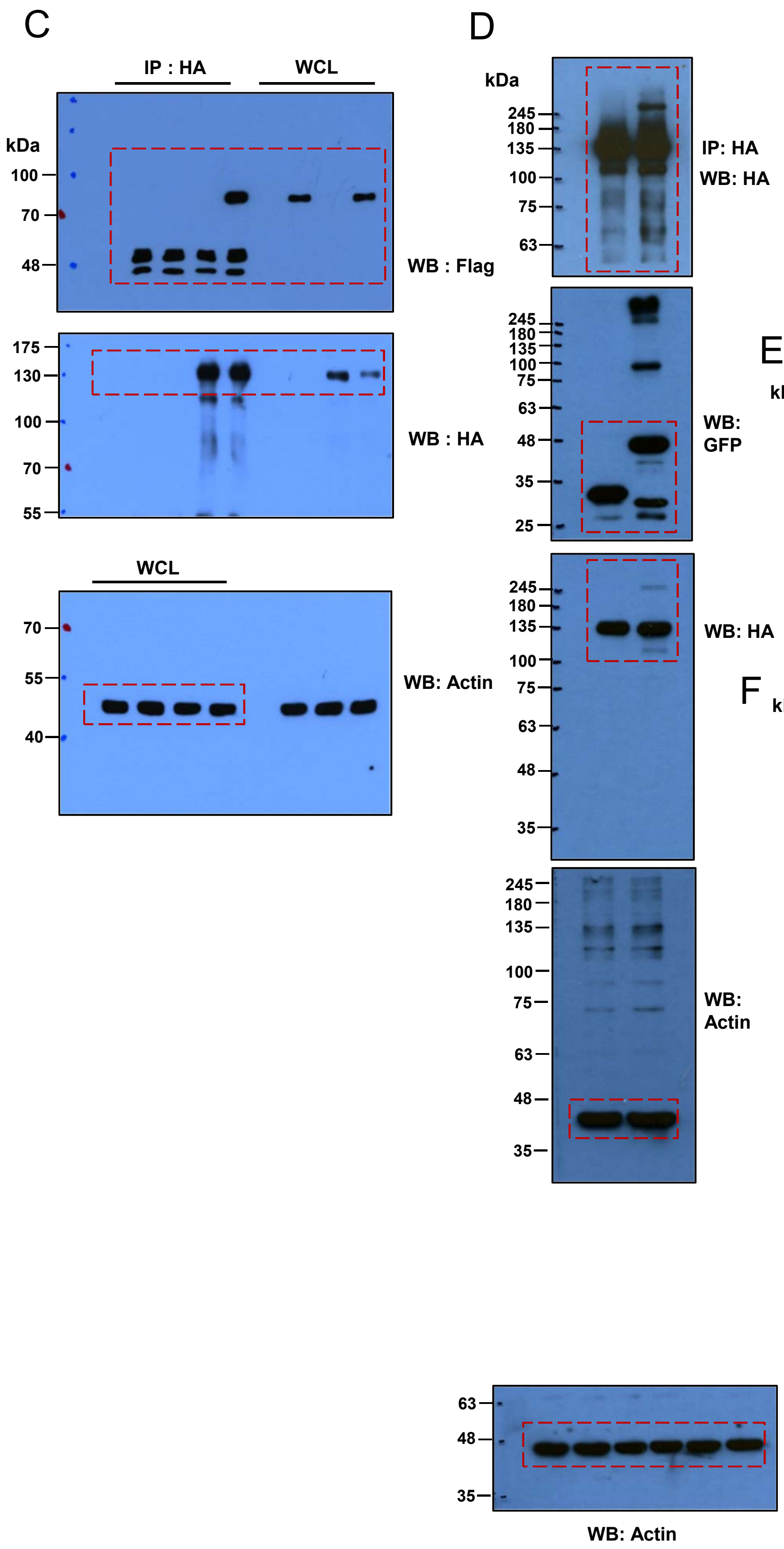

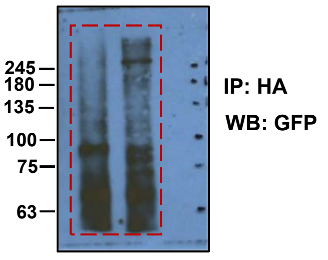

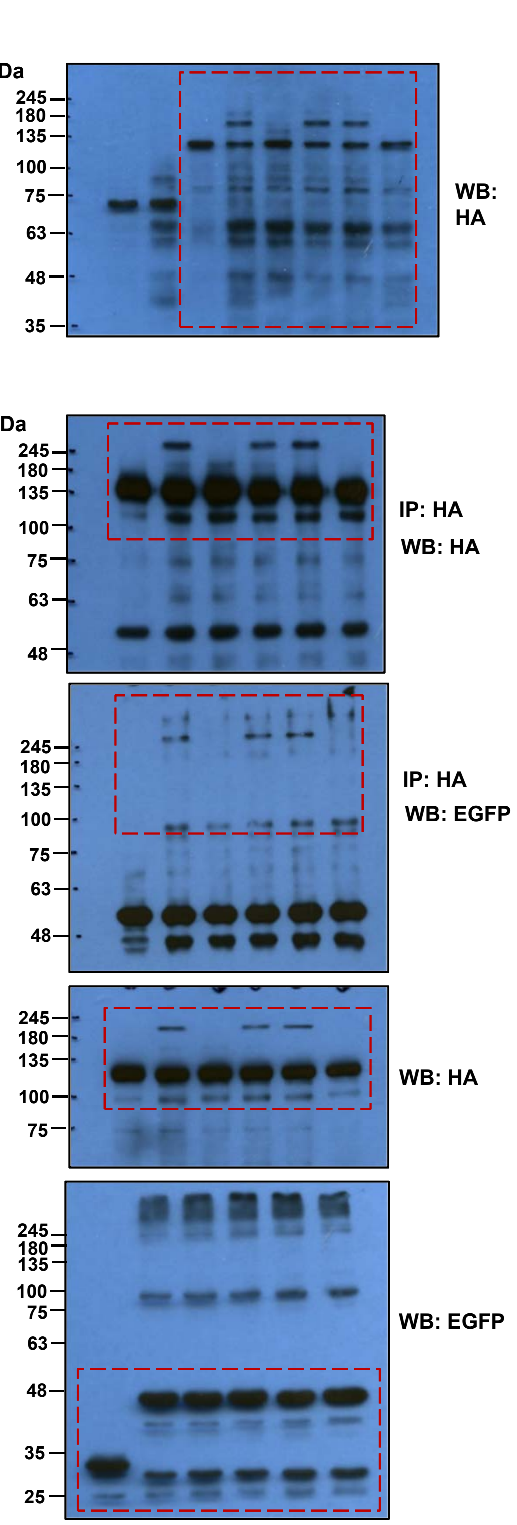

Supplementary Figure S8. Full length blots used in Figure 1. The cropped areas used in Figure 1 are shown in red boxes.

Supplemental Figure S9

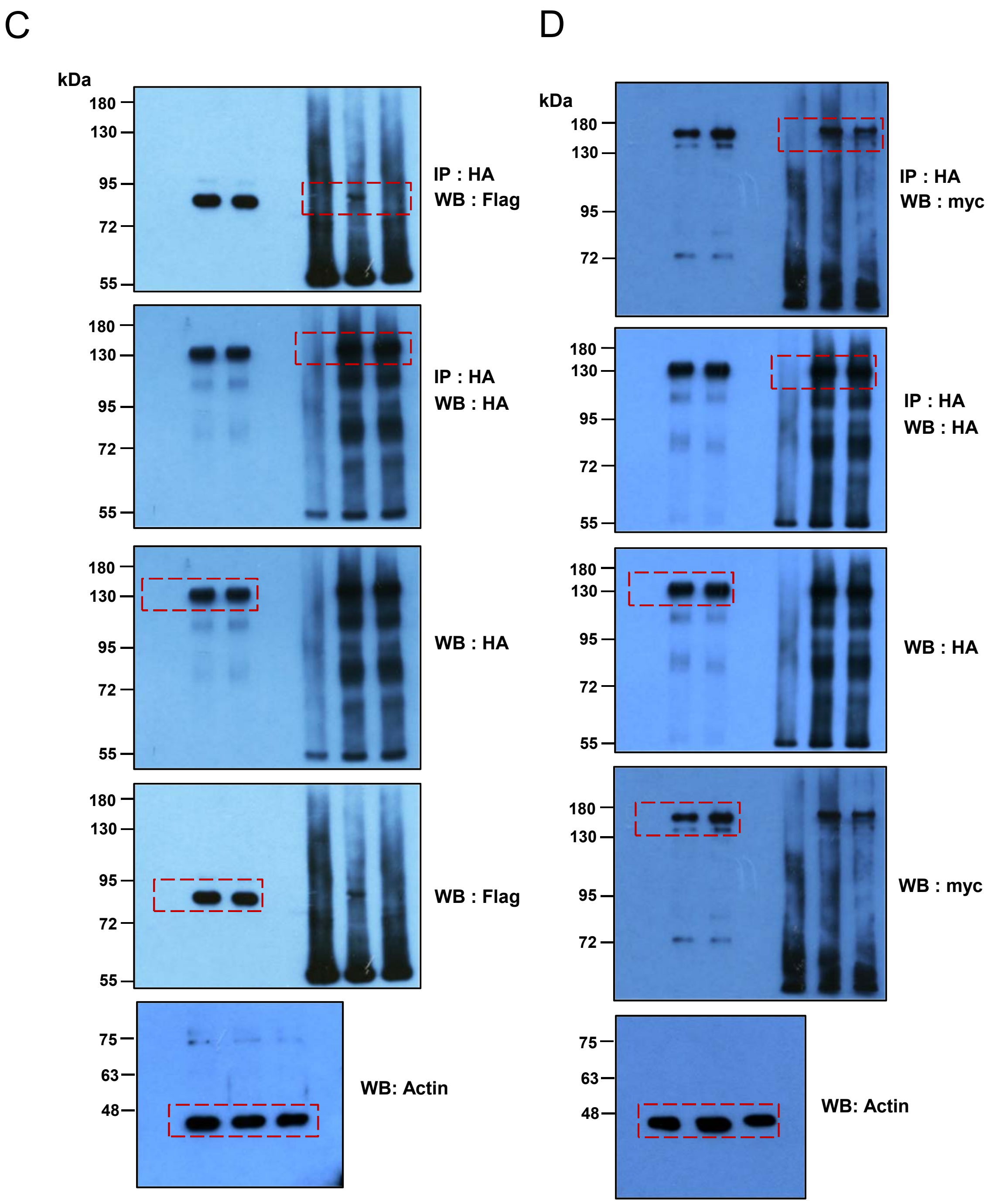

Supplementary Figure S9. Full length blots used in Figure 2. The cropped areas used in Figure 2 are shown in red boxes.

Supplemental Figure S10

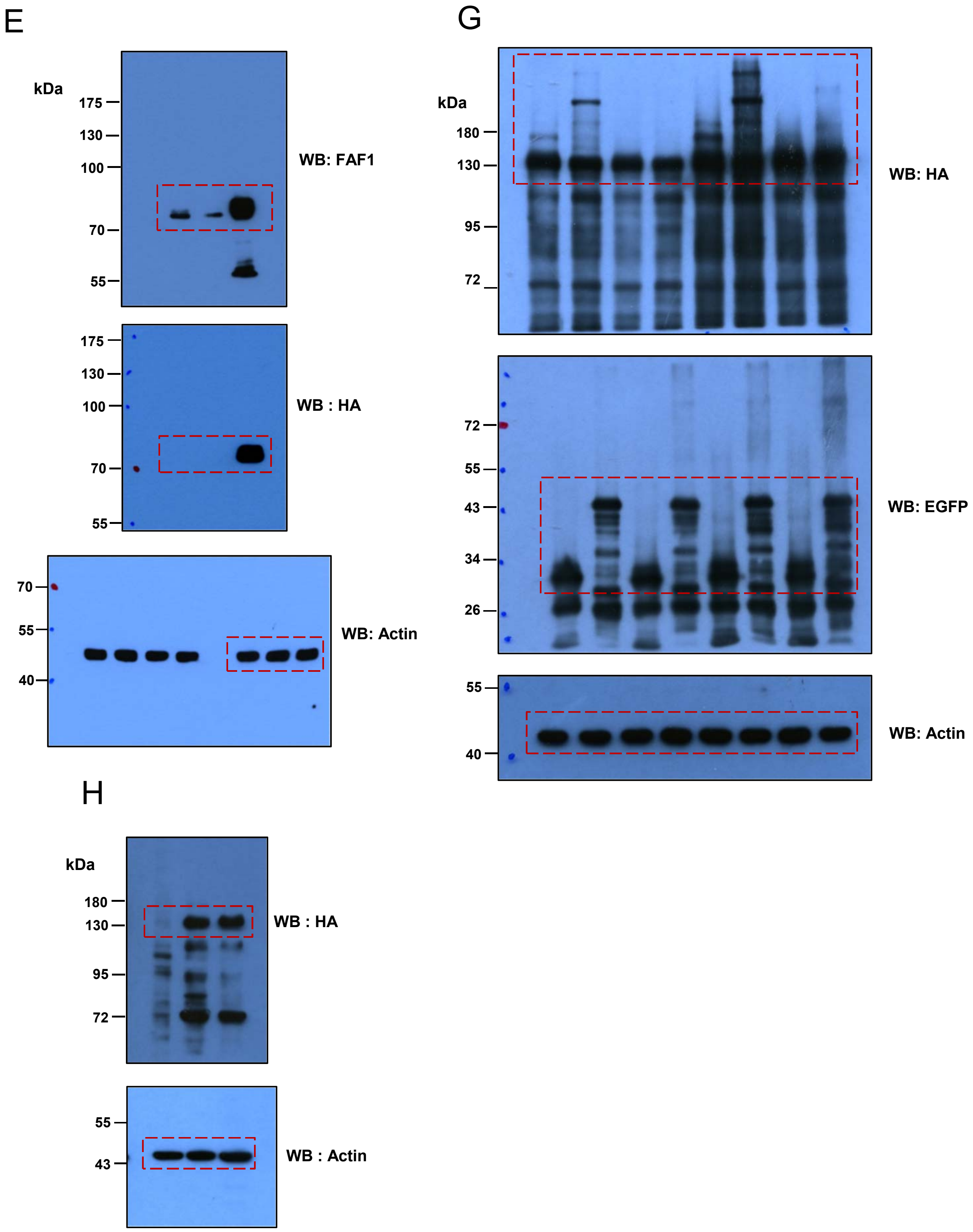

Supplementary Figure S10. Full length blots used in Figure 3. The cropped areas used in Figure 3 are shown in red boxes.

Supplemental Figure S11

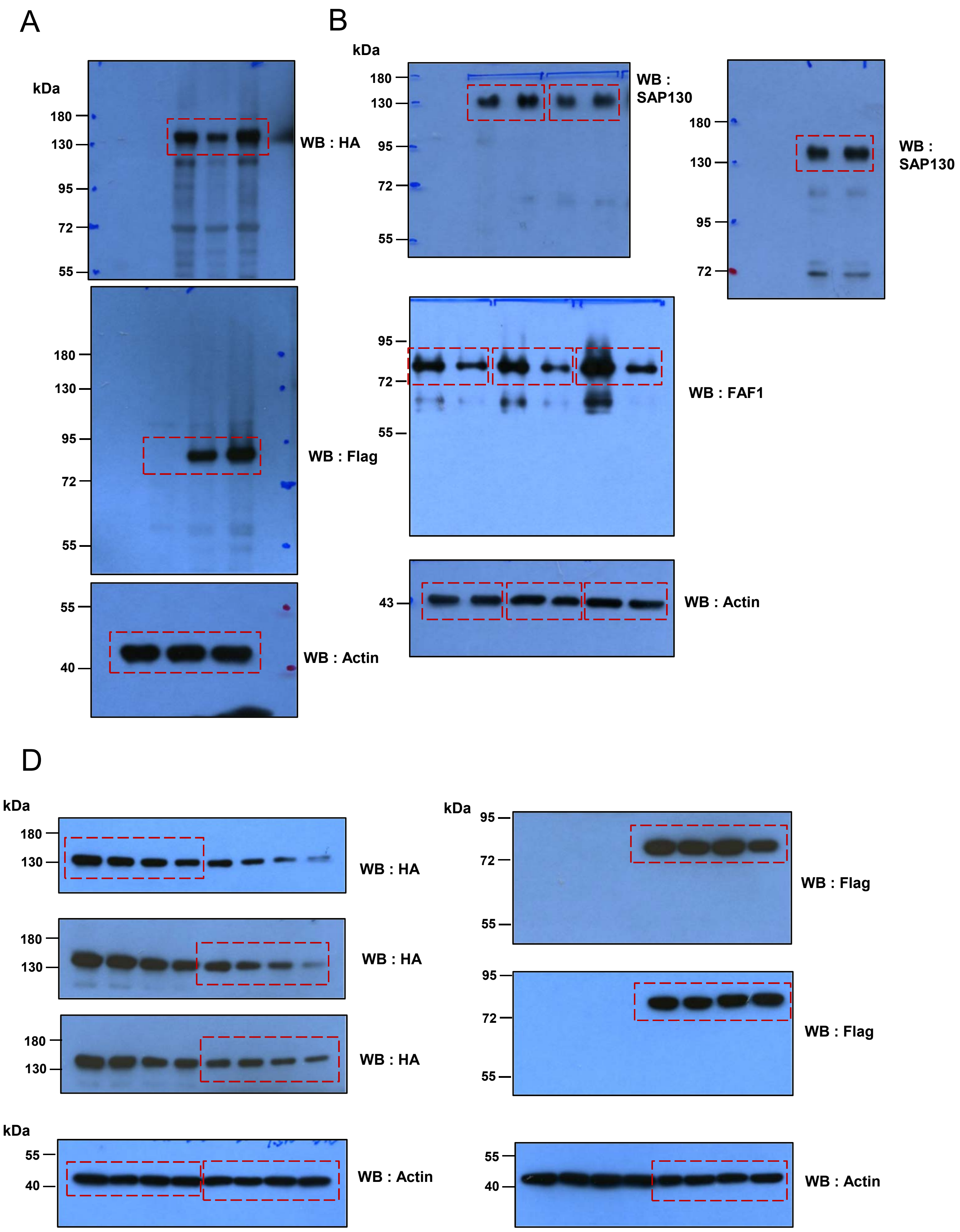

Supplemental Figure S11 (Continued)

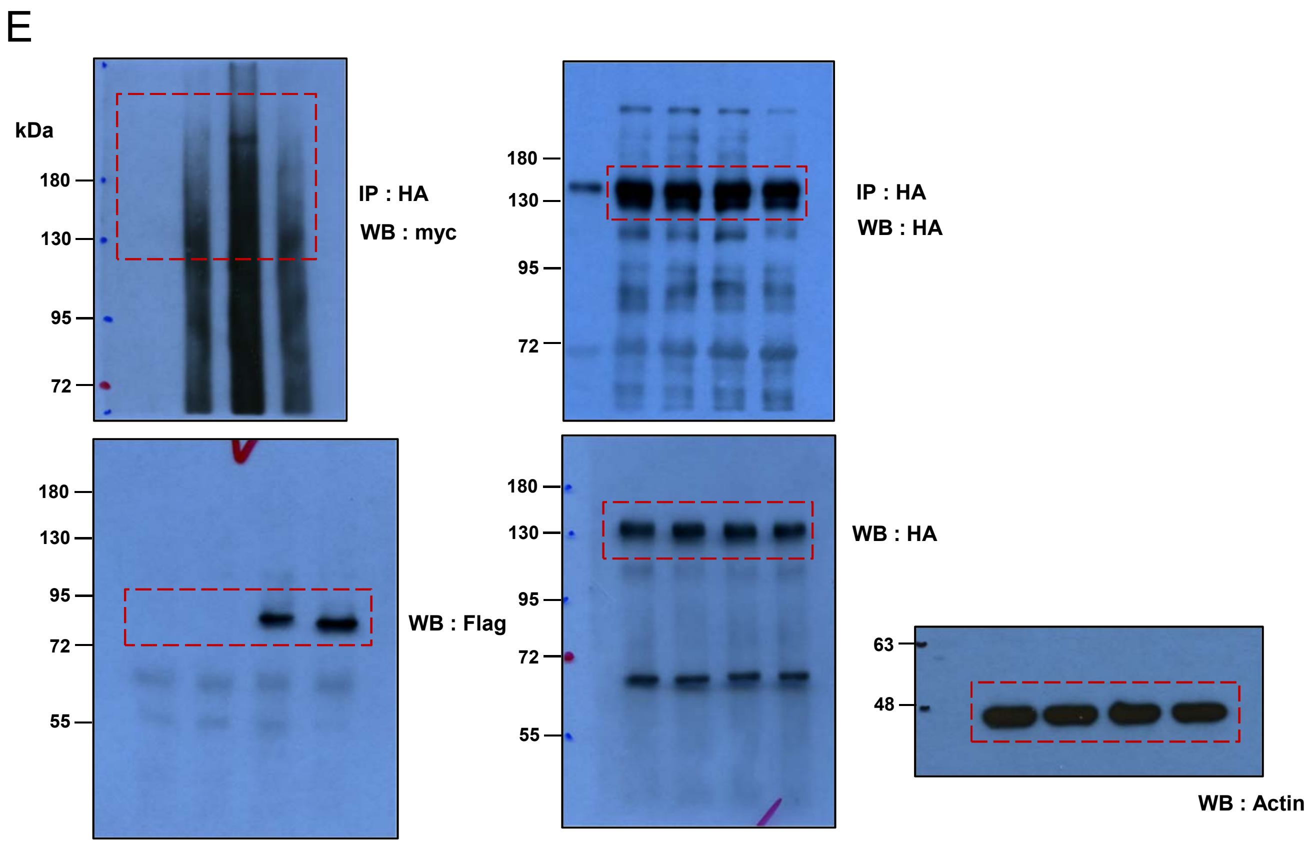

Supplementary Figure S11. Full length blots used in Figure 4. The cropped areas used in Figure 4 are shown in red boxes.

Supplemental Figure S12

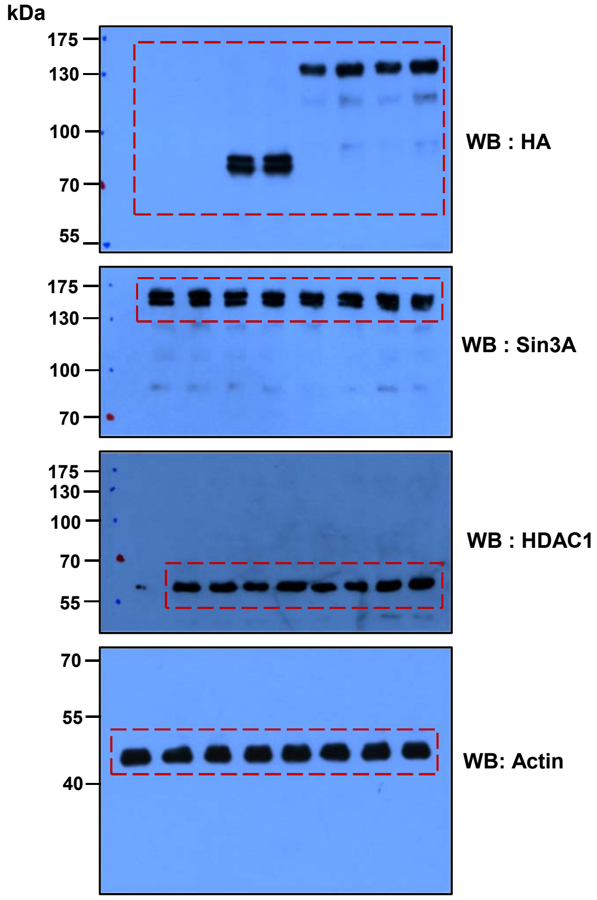

Supplementary Figure S12. Full length blots used in supplementary Figure S4. The cropped areas used in supplementary Figure S4 are shown in red boxes.

Supplemental Figure S13

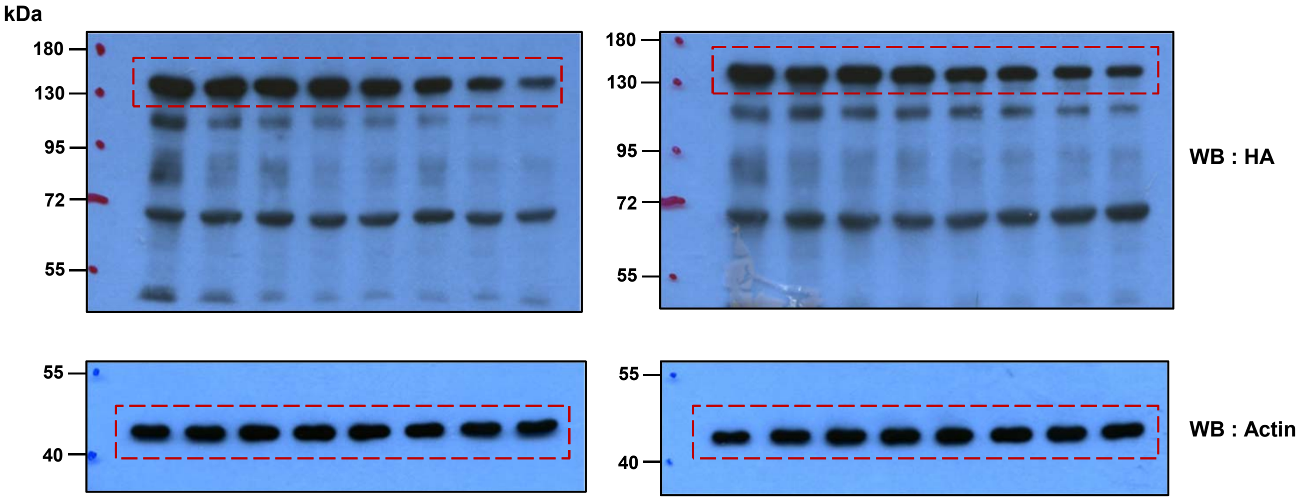

Supplementary Figure S13. Full length blots used in supplementary Figure S6. The cropped areas used in supplementary Figure S6 are shown in red boxes.
